# Peroxisome Proliferator-Activated Receptors: Experimental Targeting for the Treatment of Inflammatory Bowel Diseases

**DOI:** 10.3389/fphar.2020.00730

**Published:** 2020-05-27

**Authors:** Juan Decara, Patricia Rivera, Antonio Jesús López-Gambero, Antonia Serrano, Francisco Javier Pavón, Elena Baixeras, Fernando Rodríguez de Fonseca, Juan Suárez

**Affiliations:** ^1^ UGC Salud Mental, Instituto de Investigación Biomédica de Málaga (IBIMA), Hospital Regional Universitario de Málaga, Universidad de Málaga, Málaga, Spain; ^2^ Departamento de Endocrinología, Fundación Investigación Biomédica del Hospital Infantil Universitario Niño Jesús, Madrid, Spain; ^3^ Centro de Investigación Biomédica en Red de Enfermedades Cardiovasculares (CIBERCV) and UGC del Corazón, Instituto de Investigación Biomédica de Málaga (IBIMA), Hospital Universitario Virgen de la Victoria, Universidad de Málaga, Málaga, Spain; ^4^ Departamento de Bioquímica y Biología Molecular, Facultad de Medicina, Universidad de Málaga, IBIMA, Málaga, Spain

**Keywords:** PPARα, PPARγ, PPARβ/δ, inflammatory bowel diseases, ulcerative colitis, Crohn's disease

## Abstract

The peroxisome proliferator-activated receptors (PPARs) are a group of nuclear receptor proteins that promote ligand-dependent transcription of target genes that regulate energy production, lipid metabolism, and inflammation. The PPAR superfamily comprises three subtypes, PPARα, PPARγ, and PPARβ/δ, with differential tissue distributions. In addition to their different roles in the regulation of energy balance and carbohydrate and lipid metabolism, an emerging function of PPARs includes normal homeostasis of intestinal tissue. PPARα activation represses NF-κB signaling, which decreases the inflammatory cytokine production by different cell types, while PPARγ ligands can inhibit activation of macrophages and the production of inflammatory cytokines, such as tumor necrosis factor-alpha (TNF-α), interleukin (IL)-6, and Il-1β. In this regard, the anti-inflammatory responses induced by PPAR activation might restore physiopathological imbalances associated with inflammatory bowel diseases (IBD). Thus, PPARs and their ligands have important therapeutic potential. This review briefly discusses the roles of PPARs in the physiopathology and therapies of the most important IBDs, ulcerative colitis (UC), and Crohn's disease (CD), as well some new experimental compounds with PPAR activity as promising drugs for IBD treatment.

## Introduction

Idiopathic Inflammatory Bowel Diseases (IBDs) are chronic inflammatory disorders of the intestinal tract, the main types of which are ulcerative colitis (UC) and Crohn's disease (CD), while 10% to 15% of patients are diagnosed with some type of undetermined colitis (UC). Accumulating reports suggest that an unsuitable inflammatory response to intestinal host–microbiota–environment interactions may be associated with the onset of IBDs (Ananthakrishnan et al., 2017; Zhang et al., 2017; Zhou et al., 2017). IBDs are characterized by a chronic evolution, with severe diarrhea, abdominal pain, fatigue, and weight loss among the main symptoms, which ultimately might generate a debilitating and eventually life-threatening condition. The current treatment of IBDs is based on anti-inflammatory drugs, including 5-aaminosalicylates (5-ASA) and corticosteroids. In addition, immunosuppressant drugs or tumor necrosis factor-alpha (TNF-α) antagonists are used as complementary therapy. However, pharmacological treatment has not been optimized, and many patients need surgical procedures to remove lesioned segments of the gut. There is therefore a need for new approaches to counteract inflammatory and necrosis events that lead to destruction of the mucosa and submucosal tissues in IBD. Furthermore, new treatments could avoid the common side effects of long-term 5-ASA (Nakashima and Preuss, 2020) and corticosteroid (Buchman, 2001) treatment. Due to these limitations, new studies are immediately needed to develop new therapeutic strategies for the treatment of IBD.

The peroxisome proliferator-activated receptors (PPARs) are versatile and potent regulators of a variety of cellular functions (Evans, 1988; Mangelsdorf et al., 1995). They are part of a nuclear receptor superfamily that includes steroids and thyroid hormone receptors, retinoid receptors, and vitamin D receptors. Specifically, PPARs are a subgroup of ligand-activated transcription factors that can regulate transcriptional activity directly by two different mechanisms: 1) as a ligand-dependent transcription factor binding to DNA at the promoter region of genes with sequences known as peroxisome proliferator response elements (PPREs); and 2) as transcription factors, whereby PPARs can control gene expression by associating with activator proteins independently of PPREs (Feige et al., 2006). Therefore, PPARs can regulate sequences of many genes involved in diverse metabolic functions related to lipid and glucose homeostasis, cholesterol, and energy balance (Grygiel-Gorniak, 2014).

**Figure 1:**
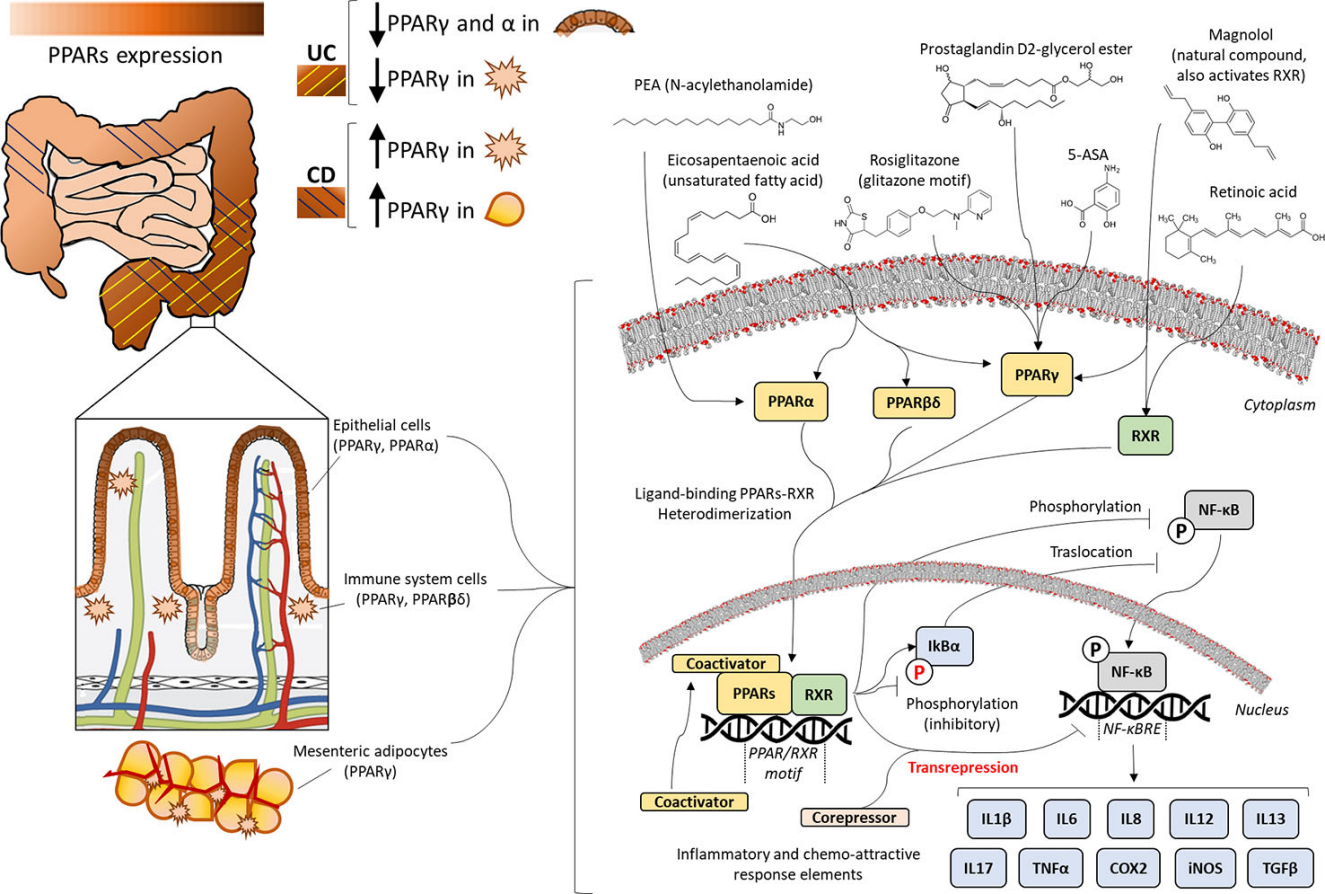
Expression and molecular mechanisms of peroxisome proliferator-activated receptors (PPARs) in inflammatory bowel disease. Presence of PPARα, PPARβ/δ, or PPARγ throughout the gastrointestinal tract is tissue-specific, as it is observed in mesenteric adipocytes, macrophages, and epithelium, most prevalently in the more differentiated layer of epithelial cells. Ulcerative colitis (UC) provokes inflammation of the colonic intestinal wall, showing decreased expression of PPARs, whereas Crohn's disease (CD) spreads to all layers and sections of the gastrointestinal tract, causing overexpression of PPARs. The anti-inflammatory efficacy of PPAR ligands (unsaturated fatty acids, 5-amino salicylic acid (5-ASA), N-acylethanolamines (NAEs), prostaglandin derivatives, Glitazones or natural compounds) is based on PPAR/retinoid-receptor X(RXR)-mediated transrepression and/or blockade of the activating phosphorylation and nuclear translocation of nuclear factor kappa-light-chain enhancer of activated B cells (NF-κB), which finally incurs the transcriptional blockage of inflammatory cytokines, chemokines, and other stress response elements, such as cyclooxygenase-2 (COX2) and inducible nitric oxide synthase (iNOS).

In this review, we will a) summarize some important aspects of PPAR biology and pharmacology, b) describe IBDs and some of the most important preclinical outcomes regarding the anti-inflammatory role of PPARs in several experimental models of IBDs, and c) discuss future therapeutic strategies against IBD. **Figure 1** summarizes the framework for understanding the mechanism of PPAR activation to modulate the pathogenesis of IBDs, as it is being reviewed in the present work.

## Peroxisome Proliferator-Activated Receptors

PPARs represent a family of ligand-activated nuclear hormone receptors (NRs) belonging to the steroid receptor superfamily. Some natural ligands include fatty acids and eicosanoids (Xu et al., 1999), but PPAR structure also reveals selective lipophilic ligand binding pockets that are able to accommodate endogenous fatty acids and their derivatives as well as different chemical compounds, such as fibrates and thiazolidinediones (TZD) used to treat metabolic disorders, including hypertriglyceridemia or type 2 diabetes (Lalloyer and Staels, 2010). Some fatty acids, including endoperoxides and acylethanolamides, might activate the three PPARs (Michalik et al., 2006). For transcriptional activity, PPARs need to heterodimerize with the retinoid-X-receptor (RXR). Thus, after activation, PPAR-RXR heterodimers can bind to specific DNA sequences called PPREs, which in turn stimulate the transcription of target genes (Berger and Moller, 2002). In consequence, depending on the type of cell, the function of PPARs can be stimulated by the presence of coactivators or inhibited by corepressors (Viswakarma et al., 2010). Due to their main roles as genetic expression modulators, PPARs also have differential tissue expression (Michalik and Wahli, 1999; Kersten et al., 2001; Michalik et al., 2006). This process can then activate transcription of various genes involved in diverse physiological and pathophysiological processes that play main roles in the pathogenesis of several chronic diseases, such as atherosclerosis (Marx et al., 1999), diabetes (Neschen et al., 2007), liver disease (Rao and Reddy, 2004), cardiovascular diseases (Takano and Kumuro, 2009), and cancer (Peters et al., 2012), involving inflammatory effects and their corresponding clinical implications (Moraes et al., 2006). In this regard, PPARs play an important role in regulating inflammation, fibrosis, and immunity (Clark, 2002). PPAR ligands can cause this to bind to the promoter sequence of target genes that participate in inflammation, modulating the proliferation, differentiation and survival of immune cells, such as macrophages, B cells, and T cells (Kostadinova et al., 2005). The mechanisms of the anti-inflammatory effects mediated by PPARs are based on the diminished proinflammatory activity of transcription factors that regulate the expression of genes responsible for inflammation, such as cytokines, adhesion molecules, and extracellular matrix proteins, and increasing the production of anti-inflammatory molecules (Kostadinova et al., 2005).

PPAR activity is triggered by endogenous compounds that modify the spatial conformation of these receptors by binding to the active ligand binding site and promoting transactivation of metabolic genes (Aagaard et al., 2011). In addition, PPARs act as transrepressors of pro-inflammatory genes as gene transcription regulators (Toyota et al., 2017). However, the simple view of ligands as “activators,” i.e., “agonists,” has recently been challenged by studies identifying compounds capable of acting as antagonists (compounds without intrinsic activity that interact with the binding site, preventing activation induced by agonists) or inverse agonists (compounds with intrinsic activity capable of eliciting PPAR repression) (Brust et al., 2018; Zheng et al., 2018).

Since clinical experience and research have been focused on PPAR agonists, the present review will be centered on drugs with direct or indirect PPAR activity. However, it is important to understand that both antagonists and inverse agonists are potentially useful new drugs that eventually might have clinical utility (Savage et al., 2015).

Furthermore, numerous synthetic compounds are used for clinic therapy, such as fibrate family members and TZDs, both of which are able to bind and activate PPARα and PPARγ, respectively, which is being used to treat metabolic disorders, such as hypertriglyceridemia or type 2 diabetes.

### Subtypes of PPAR Receptors

Three isoforms of nuclear PPAR receptors are known: PPAR-alpha (PPARα), PPAR-gamma (PPARγ), and PPAR-delta (PPARδ), also known as PPAR-beta/delta (PPARβ/δ) or nuclear receptor subfamily 1, group C, member 2 (NR1C2). PPARα is highly expressed in the muscle, heart, kidney, liver, and small and large intestine, being a target of fibrate hypolipidemic drugs implicated mainly in the catabolism of fatty acids and their oxidation (Issemann et al., 1993; Schoonjans et al., 1996). Hence, PPARα agonists have a significant importance in the treatment of dyslipidemia or metabolic syndromes by decreasing triglyceride levels in plasma (Nissen et al., 2007). Furthermore, one of the most important roles is linked to glucose homeostasis and insulin resistance, which is widely studied at present (Janani and Ranjitha Kumari, 2015). The particularity of PPARα to be expressed in peripheral tissues makes it crucial in relevant metabolic pathways implicated in the physiopathology of prevalent diseases, such as diabetes, hypertension, atherosclerosis, inflammation, cancer, or neurodegeneration (Mirza et al., 2019). Oleoylethanolamide (OEA), an anorectic acylethanolamide synthesized in the intestine, has been described as an endogenous ligand for the PPARα receptor, much like other acylethanolamides, such as palmithylethanolamide (PEA) (Rodriguez de Fonseca et al., 2001; Fu et al., 2003). Either activation of PPARα by OEA or PEA (Lo Verme et al., 2005; Anton et al., 2017) or elevation of its endogenous levels through inhibitors of the acid amidase enzyme that degrades them (Solorzano et al., 2009) has been demonstrated to have anti-inflammatory properties, suggesting a potential utility of this receptor.

PPARγ may be the most widely studied PPAR isoform. To date, several studies have evaluated the role of PPARγ in major metabolic tissues and cell types, including liver, adipocytes, pancreas, macrophages, skeletal muscle, and colonocytes, among others (Willson et al., 2001). An alteration in this ligand-activated transcription factor is associated with metabolic disorders, such as atherosclerosis (Lefebvre et al., 2006), obesity (Evans et al., 2004), metabolic syndrome and dyslipidemias (Barter and Rye, 2008), type 2 diabetes (Jay and Ren, 2007), and cancer (Grommes et al., 2004). Like PPARα, the implication of PPARγ in these prevalent diseases makes it a potential target of pharmacotherapies (Ahmadian et al., 2013). There is evidence that PPARγ may also contribute to the anti-inflammatory property of polyunsaturated fatty acids (PUFAs), as n3 PUFA enhanced hepatic regulatory T (Treg) cell generation by upregulating PPARγ and transforming growth factor beta (TGF-β) expression, thus regulating inflammatory processes in the liver (Lian et al., 2015). Along these lines, PPARγ is a target of other PUFA-derived compounds and arachidonic acid (AA), such as 15-deoxy-Δ12, 14-prostaglandin J2 (15d-PGJ2), the most recently discovered anti-inflammatory eicosanoid of which numerous beneficial effects on health are known (Marion-Letellier et al., 2016). Recent studies in rat colonic inflammation support the hypothesis of cooperation among eicosanoid prostaglandin and PPARγ, correlating the levels of 15d-PGJ2 and PPARγ expression with improved symptoms (Ponferrada et al., 2007). In this sense, antinociceptive and antiedematogenic activities of fenofibrate, an agonist of PPARα, and pioglitazone, a synthetic TZD agonist of PPARγ, have also been observed (Oliveira et al., 2007).

High expression of PPARβ/δ has been reported in the skeletal muscle, liver, kidney, adipose tissue, and intestine. It also participates in lipid homeostasis, regulation of fatty acid oxidation, inflammation, and balances of blood cholesterol and glucose (Wang et al., 2003). In this regard, PPARβ/δ activation can confer protection from atherosclerosis and metabolic disease (Lee et al., 2003). In the liver, the activation of PPARβ/δ stimulates glucose uptake and gluconeogenesis inhibition, thus improving insulin resistance and hyperglycemia (Bojic and Huff, 2013). In humans, activation of PPARβ/δ has also been found to improve the sensitivity of insulin and to counteract abnormalities related to metabolic syndrome without increasing oxidative stress (Riserus et al., 2008). Although relatively little is known, PPARβ/δ-specific functions include adiposity and placentation (Barak et al., 2002).

Due to its function as an essential regulator of target genes by transcriptional activation or repression through both ligand-dependent and -independent mechanisms and its interactions with other transcription factors, the expression of PPARβ/δ is associated with an unfavorable outcomes of several human cancers (Peters et al., 2015). The role of PPAR in inflammation, differentiation, apoptosis, and other related cancer processes is mechanistically understood, and the available data actually do not clarify the real performance of PPARβ/δ in carcinogenic processes, with the data being inconclusive and controversial (Muller, 2017).

## Inflammatory Bowel Diseases

IBD is a chronic inflammatory disorder of the gut and is distinguished by two principal conditions: UC and CD (Baumgart, 2016). CD usually affects any part of the gastrointestinal tract between the mouth and the anus, including some areas that may be interspersed and that can be confused as normal mucosa when observed microscopically and macroscopically. Furthermore, the transmural inflammation is pathognomonic in CD, being able to extend from the mucosa to the serosa layer, occasionally associated with granulomas, and even able to affect other kinds of layers in the bowel wall. This transmural inflammation present in CD can lead to complications, such as fistulas, which may perforate layers and simultaneously cause structural changes in the digestive tract. While the inflammation in UC is normally restricted to the mucosal layer of the entire colon in an uninterrupted pattern, the inflammation in CD is typically confined to the mucosa layer of the colon (Abraham and Cho, 2009). Both UC and CD may affect multiple organs beyond the gastrointestinal tract, increasing the risk of suffering additional pathologies, such as liver disease or colorectal cancer (Cosnes et al., 2011). Alteration of the immune system and inadequate commensal bacteria in the bowel (De Hertogh et al., 2008) or other luminal antigens (Baumgart and Carding, 2007) likely result in a relapsing chronic inflammatory state in humans. The origins of these enteropathies are unknown, although current hypotheses suggest that these diseases are related to a dysregulated immune response of the mucosa by nonidentified constituents of the intestinal microbiota (Shanahan, 2001) in a genetically susceptible host (Blumberg and Strober, 2001; Ananthakrishnan, 2015). There is even a hypothesis that deworming is positively correlated and early-childhood parasite infections are negatively correlated with immunologic disease, and children living in extremely hygienic environments with infrequent exposure to parasitic infections can adversely affect immune development, predisposing children to develop autoimmune diseases, such as IBD, in adult life (Moreels and Pelckmans, 2005).

The development of IBD may involve proinflammatory molecules, such as TNF-α, interleukin (IL)-1 beta (IL-1β), IL-6, interferon-gamma (IFN-γ), and IL-12, which are highly expressed and have important roles in mediating immune inflammatory responses (Neurath, 2014). Likewise, potent immunoregulatory cytokines, such as IL-10 and transforming growth factor-beta (TGF-β), can be increased in the gut compared with healthy guts (Schreiber et al., 1995), along with IL-17 expression, which is expressed by most patients with IBD (Sutherland et al., 2014).

Currently, the therapies for IBD are to prevent relapses and improve quality of life of patients. Research into IBD pathogenesis is focused on the development of therapies, including oral or rectal 5-ASA (Travis and Jewell, 1994), mesalazine, balsalazide sulfasalazine, olsalazine (Le Berre et al., 2019); traditional corticosteroids, such as prednisone, hydrocortisone, budesonide, prednisolone, dexamethasone (Damiao et al., 2019) as anti-inflammatory agents; antibiotic therapy that modulates the gut flora; and immunosuppressive agents that modulate the effects of exaggerated immune responses (Talley et al., 2011). Other promising treatments for IBD include herbal therapies because of their properties on epithelial proliferation and barrier integrity by restoring a state of microbiota homeostasis and inhibiting immune reaction (Morshedzadeh et al., 2017) and plant-derived alkaloids because of their potential antioxidant and anti-inflammatory properties (Peng et al., 2019). Biological therapies with anti-TNF-α monoclonal antibodies are also under development for IBD, including Infliximab, which is the most often used (Hemperly and Vande Casteele, 2018). Infliximab is a potent biological anti-inflammatory agent capable of diminishing the effect of TNF-α and inducing apoptosis of activated lymphocytes. However, the clinical response to this treatment is only approximately 65%, and it is associated with potential risk of infections-sepsis, lupus-like syndrome, and infusion-related reactions (Talley et al., 2011). 5-ASA is a well-known anti-inflammatory agent, but the molecular mechanism underlying its gastrointestinal effects on IBD remains unknown (Williams et al., 2011). The latest reports have shown that some adipokines, such as leptin, adiponectin, visfatin, chemerin, and retinoid binding protein (RBP4), play a role in the systemic immune responses observed in IBD patients (Zhao et al., 2017). Moreover, an imbalance between cytokines and adipokines, such as leptin and adiponectin, induced by hypercaloric diets or obesity could affect the gut microbiome, prompting an IBD disease process (Kreuter et al., 2019).

IBDs are characterized by high levels of cytokines, contributing to inflammatory processes, such as TNF-α, IL-6, IL-8, and chemokines, such as chemokine (C-X-C motif) ligand 2 (CXCL2), chemokine (C-X-C motif) ligand 3 (CXCL3), and chemokine (C-X3-C motif) ligand 1 (CX3CL1) in colon tissues (Neurath, 2014). These signals are all triggered by Nuclear Factor kappa-light-chain-enhancer of activated B cells (NF-κB) and c-Jun NH2-terminal kinase (JNK)/p38 mitogen-activated protein kinase (MAPK) pathway activation. PPARs are important for IBD because many of these signaling pathways are downregulated by the activation of PPARγ (Dubuquoy et al., 2002). Although the mechanism by which PPARγ acts on the pathogenesis of IBD has not been clarified, it may involve cyclooxygenase-2 (COX2) and NF-κB modulation, resulting in inhibition of monocyte inflammatory cytokines and reactive oxygen species (ROS). Additionally, PPARγ agonists can suppress the proinflammatory cytokines produced by monocytes, which is beneficial in chronic diseases where combined treatment with nonsteroidal anti-inflammatory drugs (NSAIDs) can be given at lower doses to minimize the side effects of long-term therapies (Jiang et al., 1998).

## Role of PPARs in the Normal and Inflamed Bowel

PPAR regulation is not fully understood, but it is known that PPARγ mRNA expression is negatively influenced by a fasting state or long-term hypocaloric diet, while it is positively affected by obesity and high fat diets with a high ratio of fatty acids (Tomas et al., 2016). In addition, an important relationship has been demonstrated between intestinal microbiota and PPARγ actions in the gut (Peyrin-Biroulet et al., 2010). Interestingly, significant downregulation of both PPARα and PPARγ mRNA expression has been found in the colonic mucosa (epithelium and lamina propria) in active UC patients at disease onset, which would implicate it in the pathophysiology of human colonic inflammation (Yamamoto-Furusho et al., 2014). Furthermore, inducible Oxide Nitric Synthase (iNOS) mRNA is overexpressed, an important pro-inflammatory mediator and nitric oxide species producer in the bowel (Cross and Wilson, 2003) that is actively controlled by PPARα, whose agonists are able to enhance its degradation (Paukkeri et al., 2007). PPARα is mainly expressed in the human colonic epithelium (Suarez et al., 2012); however, it is not expressed in immune cells of the lamina propria (Huin et al., 2000). The correlation between PPARα mRNA down-regulation in the UC mucosa and PPARα protein down-regulation in the UC epithelium demonstrates the importance of PPARs in the bowel.

PPARγ is highly expressed in the bowel (Fajas et al., 1997). Genomic descriptions of intestinal epithelial cells under PPAR agonist stimulation, as demonstrated in mice, showed the possible roles of this receptor in the gut (Bertin et al., 2013). It was reported that inadequate expression of PPARγ in intestinal epithelial cells can alter mucosal immune responses in experimental IBD (Hontecillas et al., 2011). Thus, PPARγ is involved in metabolic homeostasis and function of the intestinal epithelium; in the presence of its agonists, PPARγ can upregulate the activity of target genes (Ahmadian et al., 2013). Recently, both synthetic and natural PPARγ agonists were reported to increase the expression and activity of lactase *in vitro* and *in vivo*, supporting the importance of PPARγ activation in lactose metabolism in the intestinal epithelium (Fumery et al., 2017).

Colonization of the human gastrointestinal tract by microorganisms is established immediately at birth, and the maturation process starts when commensal bacteria contact host cells through receptors and induce signaling pathways that activate transcription of nuclear factors, such as PPARs. In this sense, the transmission of *Enterococcus faecalis* from mother to child can be regulated and activated by PPARγ in primary murine colonic epithelial cells (Are et al., 2008). This kind of study supports the concept that PPARγ in microbiota contributes to the mechanisms of initial homeostasis closely related to postnatal endocrinological development.

Adipose tissue and the large intestine are other main tissues capable of expressing PPARγ (Fajas et al., 1997). Significant interest in the biological consequences of PPARγ activation in the colon is based on its differentiating and anti-proliferative effects in adipose tissue (Chawla et al., 1994), as well as its therapeutic potential in chemoprevention of colorectal neoplasia (Saez et al., 1998; Sarraf et al., 1998). The role of PPARγ in the colon is revealed in part by the cell- and tissue-specific expression of the receptor, with high expression in colon tissue, perhaps even higher than in adipose tissue, in both rodents and humans. Furthermore, higher expression of PPARγ is described mainly in the distal colon than the small intestine and proximal colon (Lefebvre et al., 1999). Perhaps this is because PPARγ expression is mainly located in the most differentiated epithelial cells of the colon (Mansen et al., 1996; Brockman et al., 1998). Studies with cultured colon cells after differentiation are consistent with the localization of PPARγ in this tissue (Kitamura et al., 1999; Huin et al., 2002). Therefore, PPARγ expression, and its overall activation, is associated with a differentiated phenotype in cells of the intestine.

## Ulcerative Colitis and the Role of PPARs in the Inflammatory Response Associated with Disease

**Figure 2:**
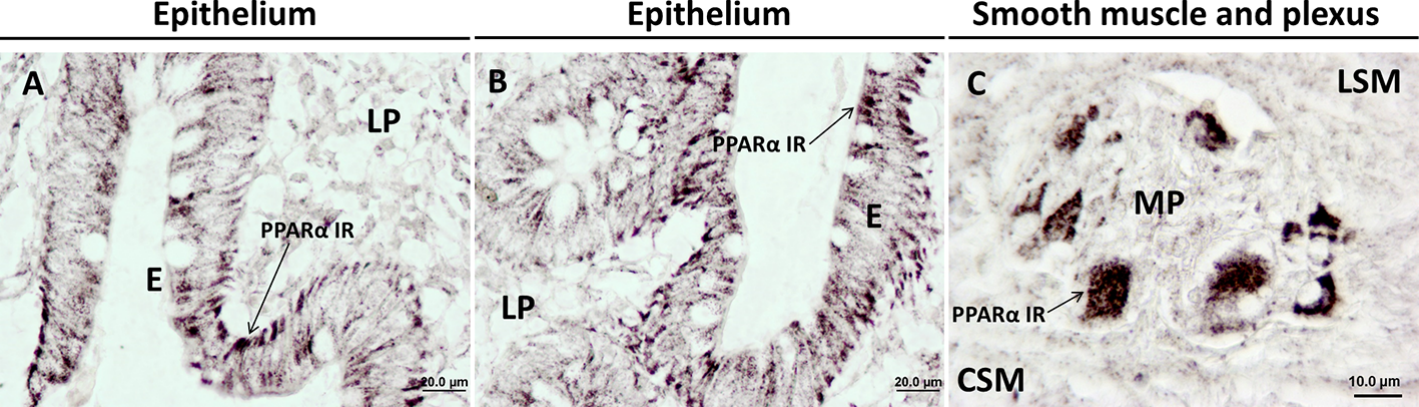
Immunohistochemical expression showing the presence and distribution of PPARα in healthy human colonic tissue. PPARα is mainly expressed in colonic epithelial cells; **(A, B)** and ganglia cells of the myenteric plexus; **(C)**. CSM, circular smooth muscle; E, epithelium; LP, lamina propria; LSM, longitudinal smooth muscle; MP, myenteric plexus. Materials and methods are described in Suarez et al. (2012).

UC is the most common form of IBD (Danese and Fiocchi, 2011). It presents as a relapsing chronic disease that involves inflammation of the colonic tissue caused by a complex combination and interaction of both genetic and environmental factors (Strober et al., 2007; Ananthakrishnan et al., 2017). The exacerbated immune response present in CD and that may contribute to inflammation includes pro-inflammatory factors, such as cytokines, reactive oxygen and nitrogen species, eicosanoids, and platelet-activating factors, among others (Sartor, 1997; Fiocchi, 1998). Currently, the therapeutic strategies for CD in humans, and in general for IBDs, include nonsteroidal anti-inflammatory drugs (e.g., sulfasalazine, mesalamine) (Ford et al., 2011a) glucocorticoids (e.g., prednisone or prednisolone, budesonide) (Lichtenstein et al., 2006), immunosuppressants (e.g., azathioprine, 6-mercaptopurine, methotrexate) (Khan et al., 2011a), antibiotics (e.g., antimycobacterial drugs, metronidazole) (Khan et al., 2011b), and anti–TNF-α antibody therapies (e.g., infliximab, adalimumab, etanercept, certolizumab) (Ford et al., 2011b). While PPARs have a well-established role in inflammation (Clark, 2002), the specific contribution of PPARs to the UC intestinal epithelium is actively under investigation (**Figure 2**; Suarez et al., 2012). Both PPARα and PPARγ are highly expressed in epithelial cells and macrophages of the intestinal and colonic mucosa (Braissant et al., 1996; Mansen et al., 1996; Huin et al., 2000). Analysis by RT-PCR, Western blot, and immunohistochemical approaches in the colon of UC patients showed decreased PPARγ mRNA and protein compared with healthy controls (Dubuquoy et al., 2003). Yamamoto-Furusho et al. (2011) also reported reduced mRNA expression of PPARγ in the mucosa of active UC compared with patients with UC in remission, suggesting a negative correlation between PPARγ and UC progression.

### Animal Models for Studying IBDs

Rodents and humans share approximately 99% of genes, showing significant similarities in the physiology of organs, metabolic processes, and pathogenesis of different diseases. Rodents are excellent model organisms thanks to their relatively small size and short generation time. To discuss the experimental data that establish a potential contribution of PPARs to the pathophysiological changes associated with IBD, we must understand the different preclinical models used to investigate this complex human disease. There are several rodent models of drug-induced IBD. The most commonly used models are dextran sodium sulphate-induced colitis (DSS-induced colitis) (Shimizu et al., 2009), trinitrobenzene sulfonic acid-induced colitis (TNBS-induced colitis) (Neurath et al., 2000), dinitrobenzene sulfonic acid-induced colitis (DNBS-induced colitis) (Dieleman et al., 1998), intra colonic acetic acid instillation-induced colitis (AA-induced colitis) (MacPherson and Pfeiffer, 1978), and oxazolone-induced colitis (OXA-induced colitis) (Boirivant et al., 1998).

One of the earliest models of spontaneous intestinal inflammation in mice is genetic IL-10 deficiency (IL-10 knockout mice) (Kuhn et al., 1993). These animal models allow the study of new approaches against IBD, as the disease can develop in them without the limitations associated with human studies.

For research and preclinical studies, genetically modified mice are commonly used, as they represent a mammalian model in which specific mouse genes can be replaced with their human equivalents. In this line, a PPARα-null mouse (PPARα knockout mice) was developed to study alterations associated with the loss of function of this receptor and the potential implication of its ligands in the regulation of metabolism (Lee et al., 1995). PPARγ-null mice have also been generated (Barak et al., 1999). Only heterozygous PPARγ knockout mice are viable; however, they have been very useful for the development of several novel compounds, which might be potential modulators of PPARγ.

With either model, combined or separate, it is possible to design new studies that may include different variables and combinations, serving as an effective tool in the search for new therapies for IBD.

### Role for PPARγ in Ulcerative Colitis

The average expression of PPARγ in the small intestine and colon is higher than that observed in other organs (Saez et al., 1998; Braissant and Wahli, 1998; Michalik and Wahli, 1999), and PPARγ expression in the colonic epithelium of UC patients is lower than controls (Lefebvre et al., 1999; Dubuquoy et al., 2003). Similar deficiencies in PPARγ expression were observed in macrophages of the lamina propria of DSS-induced colitis mice (Katayama et al., 2003). This impairment of PPARγ expression in colonocytes may be promoted by a deficiency of cortisol production (Bouguen et al., 2015). These findings suggest that chronic inflammation and/or genetic susceptibility to inflammation could be caused by decreased PPARγ expression in the colon. This hypothesis is also supported by the results obtained in studies with PPARγ-deficient mice that were highly predisposed to injuries associated with ischemia/reperfusion lesions (Nakajima et al., 2001).

Even though UC pathogenesis mechanisms are still unknown, several studies have proposed the beneficial effects of PPARγ agonists for diminution of colonic inflammation (Dworzanski et al., 2010; Celinski et al., 2011), with many reports showing PPARγ activation in intestinal tissues and its link with an anti-inflammatory role in UC (Su et al., 1999; Desreumaux et al., 2001; Nakajima et al., 2001; Wada et al., 2001; Katayama et al., 2003). At the beginning, thiopurines were commonly used to treat symptoms of UC patients (Bean, 1962), but the classic treatment for maintenance of remission from mild to moderate UC consists of 5-ASA (Wang et al., 2016a), with Mesalazine as the most used therapy (Thomson et al., 1995). In this sense, there is evidence to suggest that the anti-inflammatory effect of 5-ASAs in the colon is mediated by PPARγ (Ricote et al., 1998; Delerive et al., 2001; Rousseaux et al., 2005; Dubuquoy et al., 2006). Previous studies using animal models of irradiation-induced intestinal inflammation showed that 5-ASA is capable of inducing PPARγ expression, promoting its translocation to the nucleus of intestinal cells (Linard et al., 2008).

Synthetic PPARγ ligands, such as TZDs, are also capable of inhibiting the activation of homodimeric and heterodimeric complexes of NF-κB family members, thus strongly attenuating the immune response and diminishing the gene expression of pro-inflammatory IL-8 in colon cancer cell lines. TZDs, such as pioglitazone (Takagi et al., 2002), troglitazone (Su et al., 1999), and rosiglitazone (Su et al., 1999; Cuzzocrea et al., 2003; Ramakers et al., 2007), also provide protection in mouse models of colitis or colon cancer cell lines, suggesting that colonic PPARγ may be a potential therapeutic approach against UC in humans. Desreumaux et al. (2001) reported that both heterozygote PPARγ (Pparγ^1/_^) and retinoid X (Rxrα^1/_^) receptor-deficient mice displayed increased vulnerability to TNBS-induced colitis. As a result, synergistic activation of the RXR/PPARγ heterodimer was established by the PPARγ agonists rosiglitazone or troglitazone and the RXR agonist retinoid, suggesting protective combined effects against colon inflammation (Desreumaux et al., 2001). Further, a recent study of DSS-induced colitis showed that the partial RXR agonist CBt-PMN was also capable of ameliorating intestinal inflammation in this mouse model of UC through both PPARβ/δ/RXR and Nur77/RXR heterodimer activation (Onuki et al., 2019).

### Role for PPARγ as a Modulator of Cytokine/Chemokine Production

Deregulated production of cytokines, such as CXCL1, CXCL2, and CXCL3, is implicated in IBD pathogenesis (Neurath, 2014). These cytokines are specifically expressed in inflamed areas of the colon (Puleston at al., 2005). A recent study (Choo et al., 2015) showed how the novel PPARγ agonist 2-hydroxyethyl 5-chloro4,5-didehydrojasmonate (J11-Cl) can increase the transcription of PPARγ-dependent inflammatory pathways and reduce the intestinal inflammation in DSS-induced colitic mice. The increased transcriptional activity of PPARγ was linked to decreased pro-inflammatory cytokine production, such as IL-6 and IL-8, chemokines, including CXCL1, CXCL2, and CXCL3 in colonic tissues, and bacterial lipopolysaccharide (LPS)-induced or TNF-α–stimulated macrophages in epithelial cells. In contrast, production of anti-inflammatory cytokines, such as IL-2 and IL-4, was increased by the PPARγ agonist J11-Cl. Therefore, the study suggests that PPARγ agonists can be effective therapeutic anti-inflammatory agents for treating UC. Many pro-inflammatory cytokines are regulated by NF-κB, which plays an essential role in regulating the immune response, while its inappropriate regulation has been associated with inflammatory and autoimmune diseases, some of which are linked to cancer (Gilmore, 2006). Thus, inhibition of the NF-κB pathway could be an effective target for IBD therapy. In this way, recent studies in DSS-induced colitis mice have shown the ability of natural compounds, such as magnolol, an active ingredient of *Magnolia officinalis* (Shen et al., 2018), and *Portulaca oleracea* extract (Kong et al., 2018), from traditional Chinese medicine, to counteract the expression of cytokines, such as TNF-α, IL-1β, and IL-12, *via* the regulation of NF-κB and PPARγ pathways. Another natural compound, Sargahydroquinoic acid isolated from *Sargassum incisifolium*, and its semisynthetic derivatives have shown agonist activity of PPARγ in *in vitro* assays (Nyambe et al., 2019). In addition, it presented dual anti-inflammatory and antioxidant effects when evaluated through *in vitro* cytotoxicity against HT-29 adenocarcinoma and Caco-2 colorectal cancer cells lines in addition to PPARγ activation. At present, there is growing interest in natural products extracted from plants or herbs, especially due to the antioxidant and anti-inflammatory properties that they may present, helping epithelial barrier function and positively regulating the intestinal microbiota. In this regard, the benefit of some natural alkaloids extracted from plants has recently been reported by reducing colonic inflammation in a variety of IBD models (Peng et al., 2019). *In vitro* assays have demonstrated that tetramethylpyrazine significantly inhibits NF-κB translocation *via* IκB-α-dependent reduction of pro-inflammatory cytokines, such as TNF-α, IL-6, and IL-8, and the generation of damaging compounds, such as ROS (Scirpo et al., 2015). In addition, the same studies in OXA-induced colitis mice indicated that treatment with Tetramethylpyrazine could improve the outcome of intestinal inflammation *via* PPARγ signaling-mediated inhibition of NF-kB signaling (Lu et al., 2014).

Recently, Yu et al. (2018) also described the capability of Tropiseron, a selective 5-HT3 receptor antagonist with anti-inﬂammatory properties used to counteract chemotherapy-induced emesis, to inhibit NF-κB. To analyze the role of PPARγ in the protective effect of Tropisetron in AA-induced colitis, both macroscopic and histopathological features of the colonic injuries were evaluated, which were considerably improved upon treatment (Rahimian et al., 2016). Likewise, the levels of TNF-α, IL-1β, nitric oxide, and malondialdehyde (MDA) were significantly decreased, while an increase of PPARγ was observed, suggesting that the protective effect of tropisetron in the colon may be mediated through PPARγ (Rahimian et al., 2016).

### PPARγ Activation by COX2 Products in Ulcerative Colitis

COX2 is also involved in colon inflammation and the pathogenesis of IBD and is mostly known as an intermediate of prostaglandin (PG) production from AA. The endocannabinoids 2-arachidonoylglycerol (2-AG) and anandamide (AEA) are sources of PGs through oxygenation by COX2 to (PGH2-G) and (PGH2-EA), respectively. Such a relationship between endocannabinoids and PGs is also found between cannabinoids and PPAR agonists (Sun and Bennett, 2007). Thus, Alhouayek et al. (2018) studied the effect of a bioactive prostaglandin derived from the oxygenation of 2-AG to PGD2-G by COX2 and a hematopoietic PGD synthase (PGDS). They reported the first dual activation of PGD2 receptor (DP1) and PPARγ by PGD2-G and its anti-inflammatory properties in a murine model of colitis. They also proposed that the PPARγ activation could be mediated by 15d-PGJ2-G, a chemical metabolite of PGD2-G that may act as a PPARγ agonist.

### Lifestyle and Nutritional Factors as a Strategy Against IBD: Role of PPARs

Though it is unknown whether the anti-inflammatory properties of exercise prevent colonic inflammation in obesity (Gleeson et al., 2011), some studies have reported that exercise may induce the generation of PPARγ ligands in the plasma, which are capable of activating PPARγ signaling within monocytes and contributing to anti‐inflammatory processes (Thomas et al., 2012). There is even evidence suggesting that moderate exercise may suppress colonic inflammation in obese patients by PPARγ modulation (Liu et al., 2015a). Regarding this, it has been observed that the upregulation of glucocorticoid-mediated PPARγ activity in the colon can suppress the expression of pro-inflammatory cytokines of exercised mice compared with sedentary mice (Liu et al., 2015b).

Diet and nutritional factors could be complementary strategies for possible interventions for uncontrolled and chronic inflammation of the intestinal mucosa in IBD. It is known that microalgae species are important sources of PUFAs, which are lipid mediators with important effects on inflammation. Avila-Roman et al. (2016) studied the effects of an oxylipin-containing lyophilized biomass from *Chlamydomonas debaryana* (Oxylipin) in TNBS-induced colitis mice. They found that oral microalga biomass administration could reduce inflammation of the intestine, showing a significant decrease of pro-inflammatory cytokines, such as TNF-α, IL-1β, IL-6, and IL-17; amelioration in altered colonic morphology; and an important inhibition of body weight loss. This product could also reduce the expression of inducible nitric oxide synthase in the colon, COX2 and NF-κB, as well as increase PPARγ expression. The nutraceutical uses of this microalga, or derived oxylipins, for the treatment of IBD should be further considered.

### PPARα in Ulcerative Colitis

There is scarce scientific information about the role of PPARα in UC. Nevertheless, a few studies in experimental models of colitis have proposed that both endogenous and exogenous PPARα ligands could also have anti-inflammatory properties (Cuzzocrea et al., 2004). PPARα expression is specific in the further differentiated epithelial cells from the lumen of both the small intestine and colon (Braissant et al., 1996; Huin et al., 2000; Dubuquoy et al., 2003). A recent study also showed that 5-ASA, in addition to interfering with the control of blood lipid levels, could have a double effect by mediating PPARγ and PPARα in the small intestine, contributing positively against intestinal inflammation (Wang et al., 2018). It has also been proposed that PPARα participates in the intestinal epithelial barrier system, where the absence of its function may enhance ileum permeability during experimental colitis, while endogenous PPARα ligands can reverse this situation through the regulation of apoptosis (Mazzon and Cuzzocrea, 2007). The PPARα exogenous agonist Wy-14643 (pirinixic acid) in an IBD mouse model significantly reduced all the inflammatory parameters (Cuzzocrea et al., 2004), including decreased production of inflammatory factors that contribute to colonic damage, such as IFN-γ, IL-1β, IL-6, and TNF-α (Azuma et al., 2010). Glucocorticoids (GC) are the most used anti-inflammatory agents in the treatment of acute and chronic inflammatory diseases. With the aim of describing the anti-inflammatory role of GC-mediated PPARα, Cuzzocrea et al. (2008) and Riccardi et al. (2009) both tested the possible synergism between PPARα ligands, such as clorfibrates, and dexamethasone, a synthetic glucocorticoid, in IBD mouse models. The results of these studies indicated a modulation and improvement of the anti-inflammatory response in IBD murine models, implicating PPARα signaling in the anti-inflammatory activity of glucocorticoids. In this way, verbascoside, a glycoside that can act as an antioxidant, antibiotic, or immunosuppressive agent, and with the capacity to inhibit histamine, AA release, and prostaglandins (Mazzon et al., 2009), was proposed as a compound that can contribute to the anti-inflammatory properties of PPARα in IBD. It has also been described that Verbascoside is able to inhibit neutrophil infiltration, intestinal permeability, and colon injury in animal models of IBD (Esposito et al., 2010).

Interestingly, parasites can have significant modulatory effects on autoimmune disorders, including IBD (Moreels and Pelckmans, 2005). Epidemiologic studies and animal model experiments have shown that a recombinant protein secreted by *Schistosoma japonicum* (*S. japonicum*) (rSj16), produced by *Escherichia coli* (*E. coli*), may have immunoregulatory effects *in vivo* and *in vitro* (Wang et al., 2017). In this study, there was a positive effect of rSj16 on DSS-induced colitis by diminishing pro-inflammatory cytokine production, upregulating immunoregulatory cytokine production, and increasing Treg percentages. These effects displayed significant changes in the expression of PPARα signaling genes implicated in the colon, showing the crucial role of PPARα pathway inhibition in DSS-induced colitis development.

### N-Acylethanolamides in Ulcerative Colitis

PPARα constitutes an anti-inflammatory signaling system whose endogenous ligands belong to the N-acylethanolamides (NAEs) (Tsuboi et al., 2018). Endocannabinoid NAEs derivatives with PPARα activity, such as oleoylethanolamide (OEA) and palmitoylethanolamide (PEA), also have anti-inflammatory properties (Lambert et al., 2002; Hansen et al., 2002), with evidence that they can protect against colonic inflammation (Massa et al., 2004). The main anti-inflammatory property of PEA, which is unable also to bind both CB1 and CB2, is mediated by PPARα (Lo Verme et al., 2005), but previous reports have revealed a synergistic activation of PPARβ/δ and PPARγ that could also contribute to PPARα anti-inflammatory activity (Paterniti et al., 2013). Another report showed an improved outcome of inflammation and intestinal permeability in a model of experimental colitis in mice treated with PEA, whose effect was mediated by increased expression of colonic TRPV1 and CB1 (Borrelli et al., 2015). Along this line, a novel PEA analogue, adelmidrol, a diethanolamide derivative of azelaic acid with anti-inflammatory and anti-nociceptive properties, was able to regulate mast cell hyperreactivity in several pathophysiological conditions (Costa et al., 2008). This drug showed important anti-inflammatory effects; nonetheless, current studies remain inconclusive as to whether the effect is due to PPARα (Cordaro et al., 2016) or PPARγ (Impellizzeri et al., 2016). However, they all agree that this molecule may represent a new pharmacological approach for chronic inflammation.

Endogenous NAEs are included in the Cannabinoid System (SEC) signaling system, and these comprise enzymes involved in endocannabinoid biosynthesis, such as N-acyl phosphatidylethanolamide-specific phospholipase D (NAPE-PLD), and enzymes involved in its hydrolysis, such as fatty acid amide hydrolase (FAAH) and N-acylethanolamide hydrolyzing acid amidase (NAAA) (Tsuboi et al., 2007). In this regard, treatment with inhibitors of FAAH or its genetic absence may reduce colonic inflammation in rodent models (D'Argenio et al., 2006; Storr et al., 2008). In addition, PPARα activation by the selective inhibition of NAAA plays a primary role in reducing inflammation in UC (Solorzano et al., 2009). In recent years, several studies have indicated that cannabinoids could be a stronger protective mediator against intestinal inflammation and colorectal cancer (Hasenoehrl et al., 2016). Activation of cannabinoid receptors (CBs) by endocannabinoids influences a great number of digestive and intestinal functions, suggesting a possible treatment with cannabinoids for these pathologies in humans (Di Sabatino et al., 2011). A recent work by Grill et al. (2019) described the circulating levels of endocannabinoids in patients with IBD, and they found increased AEA and OEA, suggesting nonspecific regulation by the Endogenous Cannabinoid System (ECS) in IBD. NAE analogues with PPAR and/or dual cannabinoid/PPAR activity would be good targets in order to restore several features where homeostasis is altered, as in the case of UC. In this way, experimental models are providing robust evidence that the anti-inflammatory role of PPARs should be clarified and determined in patients with UC.

## Crohn's Disease

The main characteristic of CD is the presence of inflammation and mucosal ulceration along the gastrointestinal tract, with particular incidence in the distal small intestine. CD is frequently accompanied by fever, abdominal pain, and bowel obstruction or diarrhea. It is known that CD pathophysiology can be influenced by genetic, environmental (Ng et al., 2012), and immunobiological factors (Baumgart and Sandborn, 2012). Patients with CD are genetically susceptible individuals to environmental factors that alter the mucosal barrier, perturb the microbiota balance of the gut, and stimulate unwelcome immune responses (Boyapati et al., 2015). CD features include discontinuous, transmural inflammation in the bowel wall and an inflammatory response associated with granulomas and lymphoid aggregates (Abraham and Cho, 2009). Current treatments include traditional anti-inflammatory agents (corticosteroids) (Greenberg et al., 1994; Rezaie et al., 2015), immunomodulators (thiopurines and methotrexate), biological agents, such as antibodies directed against TNF-α, antibiotics, and surgery (Baumgart, 2016; Vetter and Neurath, 2017). The most relevant treatments targeting PPARs in different experimental models to improve the symptoms of IBD are summarized in **Table 1**.

**Table 1:** A general overview of PPARs-interacting molecules for the treatment of IBD. Both authorized and experimental products are included.

**Authorized Therapies**				
**2-ASAs**	**Target**	**Described effect**		**Reference**
Balsalazide Mesalazine Olsalazine Sulfasalazine	PPARγ	Reducing the production of inflammatory prostaglandins by COX-2 inhibition. Inhibition of chemotactic leukotrienes by 5-LO inhibition. Inhibition of macrophage and neutrophil chemotaxis		Rousseaux et al., 2005 See Wang et al., 2016a and Wang et al., 2016b for data collection and existing review analysis.
**Monoclonal Antibodies**				
Infliximab TNF-α	TNF-α	Diminishing TNF-α effect and inducing apoptosis by activated lymphocytes. PPAR-γ expression restores.		Atreya et al., 2011 Clemente et al., 2012
**Experimental/Research Therapies**				
**Endogenous Compounds**	**Target**	**Described effect**	**Experimental model**	**Reference**
PGD2-G (prostaglandin D2-glycerol ester)	DP1 PPARγ	Descriptive report, its mechanism is already unknown	DSS-induced colitis mice	Alhouayek et al., 2018
n3-PUFA (docosahexaenoic and eicosapentaenoic acids)	PPARγ NFAT	Regulate the expression and activity of the PPARγ/NFAT signaling pathway	TNBS-induced colitis rats	Yao et al., 2017
**Natural Compounds**				
*Abelmoschus manihot* (flower extract)	PPARγ	Regulate the gut microbiota and the Th17/Treg balance	DSS-induced colitis mice	Zhang et al., 2019
Magnolol (Lignin from *Magnolia officinalis*)	PPARγ	Counteracts TNF-α, IL-1β, and IL-12 expression *via* NF-κB pathway regulation	DSS-induced colitis mice	Shen et al., 2018
Oxylipins (PUFAs *Chlamydomonas debaryana* derived)	PPARγ	Decrease the pro-inflammatory cytokines TNF-α, IL-1β, IL-6, and IL-17 or reducing the expressions of COX2 and NF-κB	TNBS-induced colitis mice	Avila-Roman et al., 2016
*Portulaca oleracea* (Aqueous extract)	PPARγ	Inhibits of pro-inflammatory cytokine release and reduces the levels of NF-κB phosphorylation	DSS-induced colitis mice	Kong et al., 2018
Sargahydroquinoic acid (Semisynthetic extract from *Sargassum incisifolium)*	PPARγ	Descriptive report, its mechanism is already unknown	HT-29 and Caco-2 colorectal human cells	Nyambe et al., 2019
Tetramethylpyrazine (Fermented cocoa beans-derived alkaloid)	PPARγ	Reduces the production of inflammatory factors TNF-α, IL-6, IL-8, and ROS by activating PPARγ signaling inhibiting NF-κB pathway	OXA-induced colitis mice Caco-2 colorectal human cells	Lu et al., 2014
Verbascoside (glycoside from *Syringa vulgaris*)	PPARα	Reduction of NF-κB level and p65 and activation of the pro-active form of metalloproteinase (MMP)-2 and pro-MMP-9 activity	DNBS-induced colitis rats DNBS-induced colitis mice Ppar-α knockout mice	Mazzon et al., 2009 Esposito et al., 2010
**NAEs**				
PEA (Palmitoylethanolamide)	CB2 GPR55 PPARα	Descriptive report. Attenuated inflammation and intestinal permeability, stimulated colonic cell proliferation, and increased colonic TRPV1 and CB1 expression	DNBS-induced colitis mice	Borrelli et al., 2015
**Synthetics TZDs**				
Pioglitazone	PPARγ	Inhibition of NF-κB activation	DSS-induced colitis mice	Takagi et al., 2002
Rosiglitazone (heterocyclic compound)	PPARγ	Inhibits of IL-6, TNF-α, and NF-κB expression and neutrophil chemotaxis	HT-29 and Caco-2 colorectal DSS-induced colitis mice DSS-induced colitis rats	Su et al., 1999 Ramakers et al., 2007 Celinski et al., 2011
Troglitazone (heterocyclic compound)	PPARγ	Inhibition of NF-κB activation	HT-29 and Caco-2 colorectal human cells DSS-induced colitis mice	Su et al., 1999
**Other Synthetic Compounds**				
Adelmidrol (PEA analogue)	PPARα CB1 CB2	Reduce NF-κB translocation, COX2, and MAPK. Release pro-inflammatory cytokines. Decreased ICAM-1 and P-selectin upregulation, Bax and intensification of Bcl-2 expression	DNBS-induced colitis mice	Cordaro et al., 2016
CBt-PMN (Triazole)	PPARβ/δ RXR	Down-regulation of pro-inflammatory cytokines TNF-α and IL-6 in colon-infiltrating monocytes	DSS-induced colitis mice	Onuki et al., 2019
Dexamethasone (glucocorticoid)	PPARα	Inhibition of pro-inflammatory cytokines, cell migration, oxidative stress, and apoptosis	DNBS-induced colitis mice Ppar-α knockout mice	Riccardi et al., 2009
GED-0507-34 Levo (5-ASA analogue)	PPARγ	Reduce the state of activation of myofibroblasts and the expression of the main pro-fibrotic molecules as TGF-β, Smad3, IL-13, CTGF, and GSK-3β	DSS-induced colitis mice	Speca et al., 2016 Di Gregorio et al., 2017
J11-Cl (Jasmonate)	PPARγ	Decreases pro-inflammatory cytokines production	DSS-induced colitis mice	Choo et al., 2015
rSj16 (recombinant secreted protein of *Schistosoma japonicum*)	PPAR-α	Descriptive report: rSj16 + PPAR-α agonist: attenuated the therapeutic effects rSj16 + PPAR-α antagonist: improve the symptoms	DSS-induced colitis mice	Wang et al., 2017
Tropisetron (indole derivative)	PPARγ 5-HT3	Inhibits NF-κB, SP, and NK1R gene transcription *via* inhibiting 5-HT3R activity and its protein expression	AA-induced colitis rats	Rahimian et al., 2016 Yu et al., 2018
Wy-14643 (pirinixic acid)	PPARα	Inhibits NF-κB transcriptional activity or decreasing the IFN-γ, IL-1β, IL-6, and TNF-α production	DNBS-induced colitis mice Ppar-α knockout mice DSS-induced colitis mice	Cuzzocrea et al., 2004 Azuma et al., 2010

### PPARγ in CD

In the colon, PPARγ is highly expressed, predominantly in the epithelial surface layer (Lefebvre et al., 1999). Instead of the very low expression of PPARγ in the epithelium of the colon in both inflamed and noninflamed tissues of UC patients, normal levels were found in the colonic mucosa of both inflamed and noninflamed states of CD patients, and the mechanism for this difference it still unknown (Dubuquoy et al., 2003). A possible explanation could be related to the regulation of PPARγ expression in epithelial cells from the gut by the lipopolysaccharide bacterial receptor TLR4, whose activation leads to inflammatory cytokine production. TLR4 is responsible for activating the innate immune system and mediating the production of cytokines necessary for the development of effective immunity of inflammatory processes when stimulated by Gram-negative bacteria in the intestine (Dubuquoy et al., 2003). Regarding UC patients, TLR4 upregulation linked to the impaired expression of PPARγ may produce a lack of tolerance to microbiota in the colon and subsequent chronic inflammation (Dubuquoy et al., 2003). It was also demonstrated that both PPARγ and TNF-α are overexpressed by adipocytes from the mesentery in patients with CD (Desreumaux et al., 1999). Considering the role of PPARγ in lipogenesis, deregulation of PPARγ expression may contribute to the hypertrophy of white adipose tissue of the mesentery. This continued effect stimulates a local inflammatory response *via* TNF-α production and generates ulceration in the mucosa of the mesenteric border in CD patients (Desreumaux et al., 1999). Moreover, PPARγ antagonizes several pro-inflammatory pathways that markedly upregulate the activity of activated macrophages. Studies of PPARγ agonists also include some therapeutic roles in other inflammatory disorders, such as atherosclerosis and rheumatoid arthritis (Ricote et al., 1998), participating in immunoregulation by controlling the responses of helper T-cells in addition to having important roles in the regulatory function of T-cells (Yamazaki et al., 2002; Yamazaki et al., 2007). The SAMP1/YitFcs mouse model of CD presents similarities to many features of human CD (Takeda et al., 1981). SAMP1/YitFcs mice present ileitis characterized by affectation of the ileum by discontinuous segmental inflammation that does not occur in the proximal small intestine or colon. The histopathologic features of ileitis in SAMP1/Fc mice include transmural inflammation with abscesses, enlargement, and branching of the crypts and changes in the epithelium that include loss of villi. In connection, PPARγ has been identified as a susceptibility gene in both the SAMP/Yit mouse and in human CD (Sugawara et al., 2005). These similarities suggest that the effect of this gene in humans may be mediated through regulation of PPARγ activity in the crypts of the small intestine.

N−3 polyunsaturated fatty acids (n3-PUFAs) are widely used in the diet due to their anti−inflammatory and immunoregulatory properties by inhibiting pro-inflammatory prostaglandin and leukotriene synthesis from the AA pathway (Marion-Letellier et al., 2013). The prolonged intake of long-chain n-3 PUFAs with the diet could be associated with a decreased risk of either UC or CD (Ananthakrishnan et al., 2014). However, the basic mechanisms have not yet been clarified. Yao et al. (2017) studied the effect of n−3 PUFAs (docosahexaenoic and eicosapentaenoic acids) on TNBS-induced colitis rats, proposing a putative mechanism *via* the PPARγ/Nuclear factor of activated T-cells (NFAT) pathway. These authors also indicated markedly decreased colonic and localized mucosal inflammation as well as gene expression of the pro-inflammatory cytokines IL-2 and IL-4. On the other hand, this study indicated an increase in PPARγ expression and a reduction in NFAT gene expression.

## Anti-Fibrotic Effects of PPARs in IBDs

Intestinal fibrosis and localized inflammation are common problems of CD (Burke et al., 2007). Fibrosis occurs in at least 30% to 40% of CD cases and can involve all intestinal layers of the gastrointestinal tract affected by the disease. Intestinal fibrosis associated with CD leads to critical luminal narrowing and obstruction and usually requires surgery. Speca et al. (2016) and Di Gregorio et al. (2017) demonstrated that the specific anti-fibrotic property of GED-0507-34 Levo, a 5-ASA analogue, depends on the specific activation of PPARγ and was able to ameliorate intestinal fibrosis in the DSS-induced colitis model. These specific PPARγ ligands, with both anti-inflammatory and anti-fibrotic activities, could be promising therapeutic approaches for IBD, including UC and CD.

Fibrosis by excessive deposition of extracellular matrix components, including collagen, can be a complication of IBD, leading to organ malfunction or failure (Wynn, 2008). The relationship between inflammation and fibrosis in IBD still remains unclear, but it has been described that PPARγ activation is able to downregulate pro-inflammatory cytokine production, such as IL-4, IL-5, and IL-6, and also inhibit profibrotic molecules, such as platelet-derived growth factor (PDGF), IL-1, and TGF-β, the main promoters of fibrosis (Vetuschi et al., 2018). Increased TGF-β1 expression has been observed in the intestinal submucosal layers of fibrotic areas in CD patients (Scharl et al., 2015). On the other hand, PPARγ agonists can decrease fibrogenesis by inhibiting the effects on TGF-β signaling (Kawai et al., 2009). Furthermore, some researchers have suggested that PPARγ has both anti-inflammatory and anti-fibrotic effects in the bowel (Speca et al., 2016; Koo et al., 2017). In this regard, natural and synthetic ligands of PPARγ have been able to improve the fibrotic condition in both preliminary clinical trials and experimental models of intestinal fibrosis (Koo et al., 2017).

## Conclusion

PPARs are highly expressed in the colon and are the primary receptors for the regulation of bacteria-induced bowel inflammation. Some studies have indicated an important role for PPARs in tumor suppression, such as colon cancer. Deep knowledge of PPAR expression in the bowel and its function in inflammation will sustain the outcome of possible therapeutic approaches if we act on those targets. In addition, the development of new ligands with therapeutic efficacy on the bowel would be a potential avenue in the search for treatments or preventions of IBD. The discovery of 5-ASA and the effects of TZDs opened the way to developing new molecules targeting PPARs, more specifically PPARγ, in the bowel. Up to now, almost 20 molecules capable of activating PPARs in epithelial cells of the gut have been developed and optimized, even families of compounds that may have more efficacy than 5-ASA for PPAR activation. This approach could also avoid the common side effects of long-term 5-ASA treatment, such as headache, nausea, abdominal pain, fever, pericarditis, and liver and kidney problems, and would also avoid risks for people with allergies to sulfonamides. Therefore, drugs capable of interacting with PPARs have attracted much attention.

Optimization of these new molecules could involve seeking improvements in affinity, efficacy, and safety. Additionally, the development of new compounds as therapies that could interact, directly or indirectly, on the PPAR pathway would avoid the use of corticosteroid therapies and their known side effects, including osteoporosis, aseptic joint necrosis, adrenal insufficiency, gastrointestinal, hepatic, and ophthalmologic effects, hyperlipidemia, growth suppression, and possible congenital malformations.

Therapeutic strategies also reside in the search of drug combinations with additive or synergistic properties *via* PPARs/RXR heterodimers. In this way, the findings of synergistic effects of PPARs, TZD, and RXR agonists should be considered. Furthermore, natural ligands present in food and commensal bacteria are of interest due to their potential to induce the expression and activation of PPARs in the colon. Currently, anti-inflammatory drugs used to treat IBD are unable to attenuate intestinal fibrosis; thus, their combined action with PPARγ agonists may be a promising therapeutic approach to treat IBD. All these described data suggest the importance of associating natural regulators and synthetic ligands of PPARs as potential drug therapies for patients affected by IBD.

## Author Contributions

JD examined the literature and considered references, analyzed, interpreted the data and wrote the manuscript. PR and AL-G examined the literature and made the figures. AS, FP, EB, FR, and JS made a critical revision of the review.

## Funding

This study was supported by the following research projects and programs: Proyecto de Investigación en Salud “PI-0139-2018” funded by Consejería de Salud y Familias, Junta de Andalucía; “DTS19/00125” funded by Instituto de Salud Carlos III (ISCIII) and co-funded by European Regional Development Fund (ERDF) “A way to make Europe”; Proyecto de Investigación en Salud “PI19/01577” funded by Instituto de Salud Carlos III (ISCIII) and co-funded by European Regional Development Fund (ERDF) “A way to make Europe”; AS and JS hold “Miguel Servet” research contracts (CP14/00173 and CPII17/00024 respectively) from the ISCIII and co-funded by European Social Fund “Investing in your future” PR holds a “Sara Borrell” research contract (CD16/00067) from the ISCIII and co-funded by European Social Fund “Investing in your future” FP holds a “Programa Nicolás Monardes” contract (C1-0049-2019) from Servicio Andaluz de Salud, Junta de Andalucía.

## Conflict of Interest

The authors declare that the research was conducted in the absence of any commercial or financial relationships that could be construed as a potential conflict of interest.

## References

[B1] Aagaard M. M. Siersbaek R. Mandrup S. (2011). Molecular basis for gene-specific transactivation by nuclear receptors. Biochim. Biophys. Acta 1812 (8), 824–835. 10.1016/j.bbadis.2010.12.018 21193032

[B2] Abraham C. Cho J. H. (2009). Inflammatory bowel disease. N. Engl. J. Med. 361 (21), 2066–2078. 10.1056/NEJMra0804647 19923578 PMC3491806

[B3] Ahmadian M. Suh J. M. Hah N. Liddle C. Atkins A. R. Downes M. (2013). PPARgamma signaling and metabolism: the good, the bad and the future. Nat. Med. 19 (5), 557–566. 10.1038/nm.3159 23652116 PMC3870016

[B4] Alhouayek M. Buisseret B. Paquot A. Guillemot-Legris O. Muccioli G. G. (2018). The endogenous bioactive lipid prostaglandin D2-glycerol ester reduces murine colitis via DP1 and PPARgamma receptors. FASEB J. 32 (9), 5000–5011. 10.1096/fj.201701205R 29630407

[B5] Ananthakrishnan A. N. Khalili H. Konijeti G. G. Higuchi L. M. de Silva P. Fuchs C. S. (2014). Long-term intake of dietary fat and risk of ulcerative colitis and Crohn's disease. Gut 63 (5), 776–784. 10.1136/gutjnl-2013-305304 23828881 PMC3915038

[B6] Ananthakrishnan A. N. Bernstein C. N. Iliopoulos D. Macpherson A. Neurath M. F. Ali R. A. R. (2017). Environmental triggers in IBD: a review of progress and evidence. Nat. Rev. Gastroenterol. Hepatol. 15 (1), 39–49. 10.1038/nrgastro.2017.136 29018271

[B7] Ananthakrishnan A. N. (2015). Epidemiology and risk factors for IBD. Nat. Rev. Gastroenterol. Hepatol. 12 (4), 205–217. 10.1038/nrgastro.2015.34 25732745

[B8] Anton M. Alen F. Gomez de Heras R. Serrano A. Pavon F. J. Leza J. C. (2017). Oleoylethanolamide prevents neuroimmune HMGB1/TLR4/NF-kB danger signaling in rat frontal cortex and depressive-like behavior induced by ethanol binge administration. Addict. Biol. 22 (3), 724–741. 10.1111/adb.12365 26857094

[B9] Are A. Aronsson L. Wang S. Greicius G. Lee Y. K. Gustafsson J. A. (2008). Enterococcus faecalis from newborn babies regulate endogenous PPARgamma activity and IL-10 levels in colonic epithelial cells. Proc. Natl. Acad. Sci. U. S. A 105 (6), 1943–1948. 10.1073/pnas.0711734105 18234854 PMC2538862

[B10] Atreya R. Zimmer M. Bartsch B. Waldner M. J. Atreya I. Neumann H. (2011). Antibodies against tumor necrosis factor (TNF) induce T-cell apoptosis in patients with inflammatory bowel diseases via TNF receptor 2 and intestinal CD14(+) macrophages. Gastroenterology 141 (6), 2026–2038. 10.1053/j.gastro.2011.08.032 21875498

[B11] Avila-Roman J. Talero E. Rodriguez-Luna A. Garcia-Maurino S. Motilva V. (2016). Anti-inflammatory effects of an oxylipin-containing lyophilised biomass from a microalga in a murine recurrent colitis model. Br. J. Nutr. 116 (12), 2044–2052. 10.1017/S0007114516004189 28025954

[B12] Azuma Y. T. Nishiyama K. Matsuo Y. Kuwamura M. Morioka A. Nakajima H. (2010). PPARalpha contributes to colonic protection in mice with DSS-induced colitis. Int. Immunopharmacol. 10 (10), 1261–1267. 10.1016/j.intimp.2010.07.007 20650341

[B13] Barak Y. Nelson M. C. Ong E. S. Jones Y. Z. Ruiz-Lozano P. Chien K. R. (1999). PPAR gamma is required for placental, cardiac, and adipose tissue development. Mol. Cell 4 (4), 585–595. 10.1016/s1097-2765(00)80209-9 10549290

[B14] Barak Y. Liao D. He W. Ong E. S. Nelson M. C. Olefsky J. M. (2002). Effects of peroxisome proliferator-activated receptor delta on placentation, adiposity, and colorectal cancer. Proc. Natl. Acad. Sci. U. S. A 99 (1), 303–308. 10.1073/pnas.012610299 11756685 PMC117556

[B15] Barter P. J. Rye K. A. (2008). Is there a role for fibrates in the management of dyslipidemia in the metabolic syndrome? Arterioscler. Thromb. Vasc. Biol. 28 (1), 39–46. 10.1161/ATVBAHA.107.148817 17717290

[B16] Baumgart D. C. Carding S. R. (2007). Inflammatory bowel disease: cause and immunobiology. Lancet 369 (9573), 1627–1640. 10.1016/S0140-6736(07)60750-8 17499605

[B17] Baumgart D. C. Sandborn W. J. (2012). Crohn's disease. Lancet 380 (9853), 1590–1605. 10.1016/S0140-6736(12)60026-9 22914295

[B18] Baumgart D. C. (2016). Crohn"s disease and ulcerative colitis: from epidemiology and immunobiology to a rational diagnostic and therapeutic approach (New York, NY: Springer Science+Business Media).

[B19] Bean R. H. (1962). The treatment of chronic ulcerative colitis with 6-mercaptopurine. Med. J. Aust. 49 (2), 592–593. 10.5694/j.1326-5377.1962.tb20590.x 13969929

[B20] Berger J. Moller D. E. (2002). The mechanisms of action of PPARs. Annu. Rev. Med. 53, 409–435. 10.1146/annurev.med.53.082901.104018 11818483

[B21] Bertin B. Dubuquoy L. Colombel J. F. Desreumaux P. (2013). PPAR-gamma in ulcerative colitis: a novel target for intervention. Curr. Drug Targets 14 (12), 1501–1507. 10.2174/13894501113149990162 23651165

[B22] Blumberg R. S. Strober W. (2001). Prospects for research in inflammatory bowel disease. JAMA 285 (5), 643–647. 10.1001/jama.285.5.643 11176874

[B23] Boirivant M. Fuss I. J. Chu A. Strober W. (1998). Oxazolone colitis: A murine model of T helper cell type 2 colitis treatable with antibodies to interleukin 4. J. Exp. Med. 188 (10), 1929–1939. 10.1084/jem.188.10.1929 9815270 PMC2212414

[B24] Bojic L. A. Huff M. W. (2013). Peroxisome proliferator-activated receptor delta: a multifaceted metabolic player. Curr. Opin. Lipidol. 24 (2), 171–177. 10.1097/MOL.0b013e32835cc949 23481229

[B25] Borrelli F. Romano B. Petrosino S. Pagano E. Capasso R. Coppola D. (2015). Palmitoylethanolamide, a naturally occurring lipid, is an orally effective intestinal anti-inflammatory agent. Br. J. Pharmacol. 172 (1), 142–158. 10.1111/bph.12907 25205418 PMC4280974

[B26] Bouguen G. Langlois A. Djouina M. Branche J. Koriche D. Dewaeles E. (2015). Intestinal steroidogenesis controls PPARgamma expression in the colon and is impaired during ulcerative colitis. Gut 64 (6), 901–910. 10.1136/gutjnl-2014-307618 25053717

[B27] Boyapati R. Satsangi J. Ho G. T. (2015). Pathogenesis of Crohn's disease. F1000Prime Rep. 7, 44. 10.12703/P7-44 26097717 PMC4447044

[B28] Braissant O. Wahli W. (1998). Differential expression of peroxisome proliferator-activated receptor-alpha, -beta, and -gamma during rat embryonic development. Endocrinology 139 (6), 2748–2754. 10.1210/endo.139.6.6049 9607781

[B29] Braissant O. Foufelle F. Scotto C. Dauca M. Wahli W. (1996). Differential expression of peroxisome proliferator-activated receptors (PPARs): tissue distribution of PPAR-alpha, -beta, and -gamma in the adult rat. Endocrinology 137 (1), 354–366. 10.1210/endo.137.1.8536636 8536636

[B30] Brockman J. A. Gupta R. A. Dubois R. N. (1998). Activation of PPARgamma leads to inhibition of anchorage-independent growth of human colorectal cancer cells. Gastroenterology 115 (5), 1049–1055. 10.1016/S0016-5085(98)70072-1 9797355

[B31] Brust R. Shang J. Fuhrmann J. Mosure S. A. Bass J. Cano A. (2018). A structural mechanism for directing corepressor-selective inverse agonism of PPARgamma. Nat. Commun. 9 (1), 4687. 10.1038/s41467-018-07133-w 30409975 PMC6224492

[B32] Buchman A. L. (2001). Side effects of corticosteroid therapy. J. Clin. Gastroenterol. 33 (4), 289–294. 10.1097/00004836-200110000-00006 11588541

[B33] Burke J. P. Mulsow J. J. O'Keane C. Docherty N. G. Watson R. W. O'Connell P. R. (2007). Fibrogenesis in Crohn's disease. Am. J. Gastroenterol. 102 (2), 439–448. 10.1111/j.1572-0241.2006.01010.x 17156147

[B34] Celinski K. Dworzanski T. Korolczuk A. Piasecki R. Slomka M. Madro A. (2011). Effects of peroxisome proliferator-activated receptors-gamma ligands on dextran sodium sulphate-induced colitis in rats. J. Physiol. Pharmacol. 62 (3), 347–356. 21893696

[B35] Chawla A. Schwarz E. J. Dimaculangan D. D. Lazar M. A. (1994). Peroxisome proliferator-activated receptor (PPAR) gamma: adipose-predominant expression and induction early in adipocyte differentiation. Endocrinology 135 (2), 798–800. 10.1210/endo.135.2.8033830 8033830

[B36] Choo J. Lee Y. Yan X. J. Noh T. H. Kim S. J. Son S. (2015). A Novel Peroxisome Proliferator-activated Receptor (PPAR)gamma Agonist 2-Hydroxyethyl 5-chloro-4,5-didehydrojasmonate Exerts Anti-Inflammatory Effects in Colitis. J. Biol. Chem. 290 (42), 25609–25619. 10.1074/jbc.M115.673046 26342083 PMC4646205

[B37] Clark R. B. (2002). The role of PPARs in inflammation and immunity. J. Leukoc. Biol. 71 (3), 388–400. 11867676

[B38] Clemente T. R. Dos Santos A. N. Sturaro J. N. Gotardo E. M. de Oliveira C. C. Acedo S. C. (2012). Infliximab modifies mesenteric adipose tissue alterations and intestinal inflammation in rats with TNBS-induced colitis. Scand J. Gastroenterol 47 (8-9), 943–950. 10.3109/00365521.2012.688213 22630819

[B39] Cordaro M. Impellizzeri D. Gugliandolo E. Siracusa R. Crupi R. Esposito E. (2016). Adelmidrol, a Palmitoylethanolamide Analogue, as a New Pharmacological Treatment for the Management of Inflammatory Bowel Disease. Mol. Pharmacol. 90 (5), 549–561. 10.1124/mol.116.105668 27625036

[B40] Cosnes J. Gower-Rousseau C. Seksik P. Cortot A. (2011). Epidemiology and natural history of inflammatory bowel diseases. Gastroenterology 140 (6), 1785–1794. 10.1053/j.gastro.2011.01.055 21530745

[B41] Costa B. Comelli F. Bettoni I. Colleoni M. Giagnoni G. (2008). The endogenous fatty acid amide, palmitoylethanolamide, has anti-allodynic and anti-hyperalgesic effects in a murine model of neuropathic pain: involvement of CB(1), TRPV1 and PPARgamma receptors and neurotrophic factors. Pain 139 (3), 541–550. 10.1016/j.pain.2008.06.003 18602217

[B42] Cross R. K. Wilson K. T. (2003). Nitric oxide in inflammatory bowel disease. Inflammation Bowel Dis. 9 (3), 179–189. 10.1097/00054725-200305000-00006 12792224

[B43] Cuzzocrea S. Pisano B. Dugo L. Ianaro A. Patel N. S. Di Paola R. (2003). Rosiglitazone and 15-deoxy-Delta12,14-prostaglandin J2, ligands of the peroxisome proliferator-activated receptor-gamma (PPAR-gamma), reduce ischaemia/reperfusion injury of the gut. Br. J. Pharmacol. 140 (2), 366–376. 10.1038/sj.bjp.0705419 12970094 PMC1574022

[B44] Cuzzocrea S. Di Paola R. Mazzon E. Genovese T. Muia C. Centorrino T. (2004). Role of endogenous and exogenous ligands for the peroxisome proliferators activated receptors alpha (PPAR-alpha) in the development of inflammatory bowel disease in mice. Lab. Invest. 84 (12), 1643–1654. 10.1038/labinvest.3700185 15492755

[B45] Cuzzocrea S. Bruscoli S. Mazzon E. Crisafulli C. Donato V. Di Paola R. (2008). Peroxisome proliferator-activated receptor-alpha contributes to the anti-inflammatory activity of glucocorticoids. Mol. Pharmacol. 73 (2), 323–337. 10.1124/mol.107.041475 17984196

[B46] D'Argenio G. Valenti M. Scaglione G. Cosenza V. Sorrentini I. Di Marzo V. (2006). Up-regulation of anandamide levels as an endogenous mechanism and a pharmacological strategy to limit colon inflammation. FASEB J. 20 (3), 568–570. 10.1096/fj.05-4943fje 16403786

[B47] Damiao A. de Azevedo M. F. C. Carlos A. S. Wada M. Y. Silva T. V. M. Feitosa F. C. (2019). Conventional therapy for moderate to severe inflammatory bowel disease: A systematic literature review. World J. Gastroenterol. 25 (9), 1142–1157. 10.3748/wjg.v25.i9.1142 30863001 PMC6406187

[B48] Danese S. Fiocchi C. (2011). Ulcerative colitis. N. Engl. J. Med. 365 (18), 1713–1725. 10.1056/NEJMra1102942 22047562

[B49] De Hertogh G. Aerssens J. Geboes K. P. Geboes K. (2008). Evidence for the involvement of infectious agents in the pathogenesis of Crohn's disease. World J. Gastroenterol. 14 (6), 845–852. 10.3748/wjg.14.845 18240341 PMC2687051

[B50] Delerive P. Fruchart J. C. Staels B. (2001). Peroxisome proliferator-activated receptors in inflammation control. J. Endocrinol. 169 (3), 453–459. 10.1677/joe.0.1690453 11375115

[B51] Desreumaux P. Ernst O. Geboes K. Gambiez L. Berrebi D. Muller-Alouf H. (1999). Inflammatory alterations in mesenteric adipose tissue in Crohn's disease. Gastroenterology 117 (1), 73–81. 10.1016/S0016-5085(99)70552-4 10381912

[B52] Desreumaux P. Dubuquoy L. Nutten S. Peuchmaur M. Englaro W. Schoonjans K. (2001). Attenuation of colon inflammation through activators of the retinoid X receptor (RXR)/peroxisome proliferator-activated receptor gamma (PPARgamma) heterodimer. A basis for new therapeutic strategies. J. Exp. Med. 193 (7), 827–838. 10.1084/jem.193.7.827 11283155 PMC2193371

[B53] Di Gregorio J. Sferra R. Speca S. Vetuschi A. Dubuquoy C. Desreumaux P. (2017). Role of glycogen synthase kinase-3beta and PPAR-gamma on epithelial-to-mesenchymal transition in DSS-induced colorectal fibrosis. PloS One 12 (2), e0171093. 10.1371/journal.pone.0171093 28207769 PMC5313173

[B54] Di Sabatino A. Battista N. Biancheri P. Rapino C. Rovedatti L. Astarita G. (2011). The endogenous cannabinoid system in the gut of patients with inflammatory bowel disease. Mucosal. Immunol. 4 (5), 574–583. 10.1038/mi.2011.18 21471961

[B55] Dieleman L. A. Palmen M. J. Akol H. Bloemena E. Pena A. S. Meuwissen S. G. (1998). Chronic experimental colitis induced by dextran sulphate sodium (DSS) is characterized by Th1 and Th2 cytokines. Clin. Exp. Immunol. 114 (3), 385–391. 10.1046/j.1365-2249.1998.00728.x 9844047 PMC1905133

[B56] Dubuquoy L. Dharancy S. Nutten S. Pettersson S. Auwerx J. Desreumaux P. (2002). Role of peroxisome proliferator-activated receptor gamma and retinoid X receptor heterodimer in hepatogastroenterological diseases. Lancet 360 (9343), 1410–1418. 10.1016/S0140-6736(02)11395-X 12424006

[B57] Dubuquoy L. Jansson E. A. Deeb S. Rakotobe S. Karoui M. Colombel J. F. (2003). Impaired expression of peroxisome proliferator-activated receptor gamma in ulcerative colitis. Gastroenterology 124 (5), 1265–1276. 10.1016/S0016-5085(03)00271-3 12730867

[B58] Dubuquoy L. Rousseaux C. Thuru X. Peyrin-Biroulet L. Romano O. Chavatte P. (2006). PPARgamma as a new therapeutic target in inflammatory bowel diseases. Gut 55 (9), 1341–1349. 10.1136/gut.2006.093484 16905700 PMC1860011

[B59] Dworzanski T. Celinski K. Korolczuk A. Slomka M. Radej S. Czechowska G. (2010). Influence of the peroxisome proliferator-activated receptor gamma (PPAR-gamma) agonist, rosiglitazone and antagonist, biphenol-A-diglicydyl ether (BADGE) on the course of inflammation in the experimental model of colitis in rats. J. Physiol. Pharmacol. 61 (6), 683–693. 21224499

[B60] Esposito E. Mazzon E. Paterniti I. Dal Toso R. Pressi G. Caminiti R. (2010). PPAR-alpha Contributes to the Anti-Inflammatory Activity of Verbascoside in a Model of Inflammatory Bowel Disease in Mice. PPAR Res. 2010, 917312. 10.1155/2010/917312 20671911 PMC2910492

[B61] Evans R. M. Barish G. D. Wang Y. X. (2004). PPARs and the complex journey to obesity. Nat. Med. 10 (4), 355–361. 10.1038/nm1025 15057233

[B62] Evans R. M. (1988). The steroid and thyroid hormone receptor superfamily. Science 240 (4854), 889–895. 10.1126/science.3283939 3283939 PMC6159881

[B63] Fajas L. Auboeuf D. Raspe E. Schoonjans K. Lefebvre A. M. Saladin R. (1997). The organization, promoter analysis, and expression of the human PPARgamma gene. J. Biol. Chem. 272 (30), 18779–18789. 10.1074/jbc.272.30.18779 9228052

[B64] Feige J. N. Gelman L. Michalik L. Desvergne B. Wahli W. (2006). From molecular action to physiological outputs: peroxisome proliferator-activated receptors are nuclear receptors at the crossroads of key cellular functions. Prog. Lipid Res. 45 (2), 120–159. 10.1016/j.plipres.2005.12.002 16476485

[B65] Fiocchi C. (1998). Inflammatory bowel disease: etiology and pathogenesis. Gastroenterology 115 (1), 182–205. 10.1016/S0016-5085(98)70381-6 9649475

[B66] Ford A. C. Achkar J. P. Khan K. J. Kane S. V. Talley N. J. Marshall J. K. (2011a). Efficacy of 5-aminosalicylates in ulcerative colitis: systematic review and meta-analysis. Am. J. Gastroenterol. 106 (4), 601–616. 10.1038/ajg.2011.67 21407188

[B67] Ford A. C. Sandborn W. J. Khan K. J. Hanauer S. B. Talley N. J. Moayyedi P. (2011b). Efficacy of biological therapies in inflammatory bowel disease: systematic review and meta-analysis. Am. J. Gastroenterol. 106 (4), 644–659, quiz 660. 10.1038/ajg.2011.73 21407183

[B68] Fu J. Gaetani S. Oveisi F. Lo Verme J. Serrano A. Rodriguez De Fonseca F. (2003). Oleylethanolamide regulates feeding and body weight through activation of the nuclear receptor PPAR-alpha. Nature 425 (6953), 90–93. 10.1038/nature01921 12955147

[B69] Fumery M. Speca S. Langlois A. Davila A. M. Dubuquoy C. Grauso M. (2017). Peroxisome proliferator-activated receptor gamma (PPARgamma) regulates lactase expression and activity in the gut. EMBO Mol. Med. 9 (11), 1471–1481. 10.15252/emmm.201707795 28947679 PMC5666307

[B70] Gilmore T. D. (2006). Introduction to NF-kappaB: players, pathways, perspectives. Oncogene 25 (51), 6680–6684. 10.1038/sj.onc.1209954 17072321

[B71] Gleeson M. Bishop N. C. Stensel D. J. Lindley M. R. Mastana S. S. Nimmo M. A. (2011). The anti-inflammatory effects of exercise: mechanisms and implications for the prevention and treatment of disease. Nat. Rev. Immunol. 11 (9), 607–615. 10.1038/nri3041 21818123

[B72] Greenberg G. R. Feagan B. G. Martin F. Sutherland L. R. Thomson A. B. Williams C. N. (1994). Oral budesonide for active Crohn's disease. Canadian Inflammatory Bowel Disease Study Group. N. Engl. J. Med. 331 (13), 836–841. 10.1056/NEJM199409293311303 8078529

[B73] Grill M. Hogenauer C. Blesl A. Haybaeck J. Golob-Schwarzl N. Ferreiros N. (2019). Members of the endocannabinoid system are distinctly regulated in inflammatory bowel disease and colorectal cancer. Sci. Rep. 9 (1), 2358. 10.1038/s41598-019-38865-4 30787385 PMC6382821

[B74] Grommes C. Landreth G. E. Heneka M. T. (2004). Antineoplastic effects of peroxisome proliferator-activated receptor gamma agonists. Lancet Oncol. 5 (7), 419–429. 10.1016/S1470-2045(04)01509-8 15231248

[B75] Grygiel-Gorniak B. (2014). Peroxisome proliferator-activated receptors and their ligands: nutritional and clinical implications–a review. Nutr. J. 13, 17. 10.1186/1475-2891-13-17 24524207 PMC3943808

[B76] Hansen H. S. Moesgaard B. Petersen G. Hansen H. H. (2002). Putative neuroprotective actions of N-acyl-ethanolamines. Pharmacol. Ther. 95 (2), 119–126. 10.1016/S0163-7258(02)00251-6 12182959

[B77] Hasenoehrl C. Taschler U. Storr M. Schicho R. (2016). The gastrointestinal tract - a central organ of cannabinoid signaling in health and disease. Neurogastroenterol. Motil. 28 (12), 1765–1780. 10.1111/nmo.12931 27561826 PMC5130148

[B78] Hemperly A. Vande Casteele N. (2018). Clinical Pharmacokinetics and Pharmacodynamics of Infliximab in the Treatment of Inflammatory Bowel Disease. Clin. Pharmacokinet. 57 (8), 929–942. 10.1007/s40262-017-0627-0 29330783

[B79] Hontecillas R. Horne W. T. Climent M. Guri A. J. Evans C. Zhang Y. (2011). Immunoregulatory mechanisms of macrophage PPAR-gamma in mice with experimental inflammatory bowel disease. Mucosal. Immunol. 4 (3), 304–313. 10.1038/mi.2010.75 21068720 PMC3049196

[B80] Huin C. Corriveau L. Bianchi A. Keller J. M. Collet P. Kremarik-Bouillaud P. (2000). Differential expression of peroxisome proliferator-activated receptors (PPARs) in the developing human fetal digestive tract. J. Histochem. Cytochem. 48 (5), 603–611. 10.1177/002215540004800504 10769044

[B81] Huin C. Schohn H. Hatier R. Bentejac M. Antunes L. Plenat F. (2002). Expression of peroxisome proliferator-activated receptors alpha and gamma in differentiating human colon carcinoma Caco-2 cells. Biol. Cell 94 (1), 15–27. 10.1016/S0248-4900(01)01178-9 12000143

[B82] Impellizzeri D. Di Paola R. Cordaro M. Gugliandolo E. Casili G. Morittu V. M. (2016). Adelmidrol, a palmitoylethanolamide analogue, as a new pharmacological treatment for the management of acute and chronic inflammation. Biochem. Pharmacol. 119, 27–41. 10.1016/j.bcp.2016.09.001 27599446

[B83] Issemann I. Prince R. A. Tugwood J. D. Green S. (1993). The peroxisome proliferator-activated receptor:retinoid X receptor heterodimer is activated by fatty acids and fibrate hypolipidaemic drugs. J. Mol. Endocrinol. 11 (1), 37–47. 10.1677/jme.0.0110037 8240670

[B84] Janani C. Ranjitha Kumari B. D. (2015). PPAR gamma gene–a review. Diabetes Metab. Syndr. 9 (1), 46–50. 10.1016/j.dsx.2014.09.015 25450819

[B85] Jay M. A. Ren J. (2007). Peroxisome proliferator-activated receptor (PPAR) in metabolic syndrome and type 2 diabetes mellitus. Curr. Diabetes Rev. 3 (1), 33–39. 10.2174/157339907779802067 18220654

[B86] Jiang C. Ting A. T. Seed B. (1998). PPAR-gamma agonists inhibit production of monocyte inflammatory cytokines. Nature 391 (6662), 82–86. 10.1038/34184 9422509

[B87] Katayama K. Wada K. Nakajima A. Mizuguchi H. Hayakawa T. Nakagawa S. (2003). A novel PPAR gamma gene therapy to control inflammation associated with inflammatory bowel disease in a murine model. Gastroenterology 124 (5), 1315–1324. 10.1016/S0016-5085(03)00262-2 12730872

[B88] Kawai T. Masaki T. Doi S. Arakawa T. Yokoyama Y. Doi T. (2009). PPAR-gamma agonist attenuates renal interstitial fibrosis and inflammation through reduction of TGF-beta. Lab. Invest. 89 (1), 47–58. 10.1038/labinvest.2008.104 19002105

[B89] Kersten S. Mandard S. Escher P. Gonzalez F. J. Tafuri S. Desvergne B. (2001). The peroxisome proliferator-activated receptor alpha regulates amino acid metabolism. FASEB J. 15 (11), 1971–1978. 10.1096/fj.01-0147com 11532977

[B90] Khan K. J. Dubinsky M. C. Ford A. C. Ullman T. A. Talley N. J. Moayyedi P. (2011a). Efficacy of immunosuppressive therapy for inflammatory bowel disease: a systematic review and meta-analysis. Am. J. Gastroenterol. 106 (4), 630–642. 10.1038/ajg.2011.64 21407186

[B91] Khan K. J. Ullman T. A. Ford A. C. Abreu M. T. Abadir A. Marshall J. K. (2011b). Antibiotic therapy in inflammatory bowel disease: a systematic review and meta-analysis. Am. J. Gastroenterol. 106 (4), 661–673. 10.1038/ajg.2011.72 21407187

[B92] Kitamura S. Miyazaki Y. Shinomura Y. Kondo S. Kanayama S. Matsuzawa Y. (1999). Peroxisome proliferator-activated receptor gamma induces growth arrest and differentiation markers of human colon cancer cells. Jpn. J. Cancer Res. 90 (1), 75–80. 10.1111/j.1349-7006.1999.tb00668.x 10076568 PMC5925976

[B93] Kong R. Luo H. Wang N. Li J. Xu S. Chen K. (2018). Portulaca Extract Attenuates Development of Dextran Sulfate Sodium Induced Colitis in Mice through Activation of PPARgamma. PPAR Res. 2018, 6079101. 10.1155/2018/6079101 29483924 PMC5816873

[B94] Koo J. B. Nam M. O. Jung Y. Yoo J. Kim D. H. Kim G. (2017). Anti-fibrogenic effect of PPAR-gamma agonists in human intestinal myofibroblasts. BMC Gastroenterol. 17 (1), 73. 10.1186/s12876-017-0627-4 28592228 PMC5463383

[B95] Kostadinova R. Wahli W. Michalik L. (2005). PPARs in diseases: control mechanisms of inflammation. Curr. Med. Chem. 12 (25), 2995–3009. 10.2174/092986705774462905 16378501

[B96] Kreuter R. Wankell M. Ahlenstiel G. Hebbard L. (2019). The role of obesity in inflammatory bowel disease. Biochim. Biophys. Acta Mol. Basis Dis. 1865 (1), 63–72. 10.1016/j.bbadis.2018.10.020 30352258

[B97] Kuhn R. Lohler J. Rennick D. Rajewsky K. Muller W. (1993). Interleukin-10-deficient mice develop chronic enterocolitis. Cell 75 (2), 263–274. 10.1016/0092-8674(93)80068-P 8402911

[B98] Lalloyer F. Staels B. (2010). Fibrates, glitazones, and peroxisome proliferator-activated receptors. Arterioscler. Thromb. Vasc. Biol. 30 (5), 894–899. 10.1161/ATVBAHA.108.179689 20393155 PMC2997800

[B99] Lambert D. M. Vandevoorde S. Jonsson K. O. Fowler C. J. (2002). The palmitoylethanolamide family: a new class of anti-inflammatory agents? Curr. Med. Chem. 9 (6), 663–674. 10.2174/0929867023370707 11945130

[B100] Le Berre C. Roda G. Nedeljkovic Protic M. Danese S. Peyrin-Biroulet L. (2019). Modern use of 5-aminosalicylic acid compounds for ulcerative colitis. Expert Opin. Biol. Ther. 20 (4), 363–378. 10.1080/14712598.2019.1666101 31498003

[B101] Lee S. S. Pineau T. Drago J. Lee E. J. Owens J. W. Kroetz D. L. (1995). Targeted disruption of the alpha isoform of the peroxisome proliferator-activated receptor gene in mice results in abolishment of the pleiotropic effects of peroxisome proliferators. Mol. Cell Biol. 15 (6), 3012–3022. 10.1128/MCB.15.6.3012 7539101 PMC230532

[B102] Lee C. H. Chawla A. Urbiztondo N. Liao D. Boisvert W. A. Evans R. M. (2003). Transcriptional repression of atherogenic inflammation: modulation by PPARdelta. Science 302 (5644), 453–457. 10.1126/science.1087344 12970571

[B103] Lefebvre M. Paulweber B. Fajas L. Woods J. McCrary C. Colombel J. F. (1999). Peroxisome proliferator-activated receptor gamma is induced during differentiation of colon epithelium cells. J. Endocrinol. 162 (3), 331–340. 10.1677/joe.0.1620331 10467224

[B104] Lefebvre P. Chinetti G. Fruchart J. C. Staels B. (2006). Sorting out the roles of PPAR alpha in energy metabolism and vascular homeostasis. J. Clin. Invest. 116 (3), 571–580. 10.1172/JCI27989 16511589 PMC1386122

[B105] Lian M. Luo W. Sui Y. Li Z. Hua J. (2015). Dietary n-3 PUFA Protects Mice from Con A Induced Liver Injury by Modulating Regulatory T Cells and PPAR-gamma Expression. PloS One 10 (7), e0132741. 10.1371/journal.pone.0132741 26177196 PMC4503783

[B106] Lichtenstein G. R. Abreu M. T. Cohen R. Tremaine W. (2006). American Gastroenterological Association Institute technical review on corticosteroids, immunomodulators, and infliximab in inflammatory bowel disease. Gastroenterology 130 (3), 940–987. 10.1053/j.gastro.2006.01.048 16530532

[B107] Linard C. Gremy O. Benderitter M. (2008). Reduction of peroxisome proliferation-activated receptor gamma expression by gamma-irradiation as a mechanism contributing to inflammatory response in rat colon: modulation by the 5-aminosalicylic acid agonist. J. Pharmacol. Exp. Ther. 324 (3), 911–920. 10.1124/jpet.107.129122 18077625

[B108] Liu W. X. Wang T. Zhou F. Wang Y. Xing J. W. Zhang S. (2015a). Voluntary exercise prevents colonic inflammation in high-fat diet-induced obese mice by up-regulating PPAR-gamma activity. Biochem. Biophys. Res. Commun. 459 (3), 475–480. 10.1016/j.bbrc.2015.02.047 25701789

[B109] Liu W. X. Zhou F. Wang Y. Wang T. Xing J. W. Zhang S. (2015b). Voluntary exercise protects against ulcerative colitis by up-regulating glucocorticoid-mediated PPAR-gamma activity in the colon in mice. Acta Physiol. (Oxf) 215 (1), 24–36. 10.1111/apha.12534 26031185

[B110] Lo Verme J. Fu J. Astarita G. La Rana G. Russo R. Calignano A. (2005). The nuclear receptor peroxisome proliferator-activated receptor-alpha mediates the anti-inflammatory actions of palmitoylethanolamide. Mol. Pharmacol. 67 (1), 15–19. 10.1124/mol.104.006353 15465922

[B111] Lu Y. Zhu M. Chen W. Yin L. Zhu J. Chen N. (2014). Tetramethylpyrazine improves oxazolone-induced colitis by inhibiting the NF-kappaB pathway. Clin. Invest. Med. 37 (1), E1–E9. 10.25011/cim.v37i1.20863 24502806

[B112] MacPherson B. R. Pfeiffer C. J. (1978). Experimental production of diffuse colitis in rats. Digestion 17 (2), 135–150. 10.1159/000198104 627326

[B113] Mangelsdorf D. J. Thummel C. Beato M. Herrlich P. Schutz G. Umesono K. (1995). The nuclear receptor superfamily: the second decade. Cell 83 (6), 835–839. 10.1016/0092-8674(95)90199-X 8521507 PMC6159888

[B114] Mansen A. Guardiola-Diaz H. Rafter J. Branting C. Gustafsson J. A. (1996). Expression of the peroxisome proliferator-activated receptor (PPAR) in the mouse colonic mucosa. Biochem. Biophys. Res. Commun. 222 (3), 844–851. 10.1006/bbrc.1996.0832 8651933

[B115] Marion-Letellier R. Savoye G. Beck P. L. Panaccione R. Ghosh S. (2013). Polyunsaturated fatty acids in inflammatory bowel diseases: a reappraisal of effects and therapeutic approaches. Inflammation Bowel Dis. 19 (3), 650–661. 10.1097/MIB.0b013e3182810122 23328774

[B116] Marion-Letellier R. Savoye G. Ghosh S. (2016). Fatty acids, eicosanoids and PPAR gamma. Eur. J. Pharmacol. 785, 44–49. 10.1016/j.ejphar.2015.11.004 26632493

[B117] Marx N. Bourcier T. Sukhova G. K. Libby P. Plutzky J. (1999). PPARgamma activation in human endothelial cells increases plasminogen activator inhibitor type-1 expression: PPARgamma as a potential mediator in vascular disease. Arterioscler. Thromb. Vasc. Biol. 19 (3), 546–551. 10.1161/01.atv.19.3.546 10073956

[B118] Massa F. Marsicano G. Hermann H. Cannich A. Monory K. Cravatt B. F. (2004). The endogenous cannabinoid system protects against colonic inflammation. J. Clin. Invest. 113 (8), 1202–1209. 10.1172/JCI19465 15085199 PMC385396

[B119] Mazzon E. Cuzzocrea S. (2007). Absence of functional peroxisome proliferator-activated receptor-alpha enhanced ileum permeability during experimental colitis. Shock 28 (2), 192–201. 10.1097/SHK.0b013e318033eb29 17515853

[B120] Mazzon E. Esposito E. Di Paola R. Riccardi L. Caminiti R. Dal Toso R. (2009). Effects of verbascoside biotechnologically produced by Syringa vulgaris plant cell cultures in a rodent model of colitis. Naunyn Schmiedebergs Arch. Pharmacol. 380 (1), 79–94. 10.1007/s00210-009-0400-5 19242677

[B121] Michalik L. Wahli W. (1999). Peroxisome proliferator-activated receptors: three isotypes for a multitude of functions. Curr. Opin. Biotechnol. 10 (6), 564–570. 10.1016/S0958-1669(99)00030-0 10600688

[B122] Michalik L. Auwerx J. Berger J. P. Chatterjee V. K. Glass C. K. Gonzalez F. J. (2006). International Union of Pharmacology. LXI. Peroxisome proliferator-activated receptors. Pharmacol. Rev. 58 (4), 726–741. 10.1124/pr.58.4.5 17132851

[B123] Mirza A. Z. Althagafi I. I. Shamshad H. (2019). Role of PPAR receptor in different diseases and their ligands: Physiological importance and clinical implications. Eur. J. Med. Chem. 166, 502–513. 10.1016/j.ejmech.2019.01.067 30739829

[B124] Moraes L. A. Piqueras L. Bishop-Bailey D. (2006). Peroxisome proliferator-activated receptors and inflammation. Pharmacol. Ther. 110 (3), 371–385. 10.1016/j.pharmthera.2005.08.007 16168490

[B125] Moreels T. G. Pelckmans P. A. (2005). Gastrointestinal parasites: potential therapy for refractory inflammatory bowel diseases. Inflammation Bowel Dis. 11 (2), 178–184. 10.1097/00054725-200502000-00012 15677912

[B126] Morshedzadeh N. Rahimlou M. Asadzadeh Aghdaei H. Shahrokh S. Reza Zali M. Mirmiran P. (2017). Association Between Adipokines Levels with Inflammatory Bowel Disease (IBD): Systematic Reviews. Dig. Dis. Sci. 62 (12), 3280–3286. 10.1007/s10620-017-4806-5 29086333

[B127] Muller R. (2017). PPARbeta/delta in human cancer. Biochimie 136, 90–99. 10.1016/j.biochi.2016.10.019 27916645

[B128] Nakajima A. Wada K. Miki H. Kubota N. Nakajima N. Terauchi Y. (2001). Endogenous PPAR gamma mediates anti-inflammatory activity in murine ischemia-reperfusion injury. Gastroenterology 120 (2), 460–469. 10.1053/gast.2001.21191 11159886

[B129] Nakashima J. Preuss C. V. (2020). “Mesalamine (USAN),” (Treasure Island (FL): StatPearls). 31869178

[B130] Neschen S. Morino K. Dong J. Wang-Fischer Y. Cline G. W. Romanelli A. J. (2007). n-3 Fatty acids preserve insulin sensitivity in vivo in a peroxisome proliferator-activated receptor-alpha-dependent manner. Diabetes 56 (4), 1034–1041. 10.2337/db06-1206 17251275

[B131] Neurath M. Fuss I. Strober W. (2000). TNBS-colitis. Int. Rev. Immunol. 19 (1), 51–62. 10.3109/08830180009048389 10723677

[B132] Neurath M. F. (2014). Cytokines in inflammatory bowel disease. Nat. Rev. Immunol. 14 (5), 329–342. 10.1038/nri3661 24751956

[B133] Ng S. C. Woodrow S. Patel N. Subhani J. Harbord M. (2012). Role of genetic and environmental factors in British twins with inflammatory bowel disease. Inflammation Bowel Dis. 18 (4), 725–736. 10.1002/ibd.21747 21557397

[B134] Nissen S. E. Nicholls S. J. Wolski K. Howey D. C. McErlean E. Wang M. D. (2007). Effects of a potent and selective PPAR-alpha agonist in patients with atherogenic dyslipidemia or hypercholesterolemia: two randomized controlled trials. JAMA 297 (12), 1362–1373. 10.1001/jama.297.12.1362 17384435

[B135] Nyambe M. N. Koekemoer T. C. van de Venter M. Goosen E. D. Beukes D. R. (2019). In Vitro Evaluation of the Phytopharmacological Potential of Sargassum incisifolium for the Treatment of Inflammatory Bowel Diseases. Medicines (Basel) 6 (2), E49. 10.3390/medicines6020049 PMC6631261 30959861

[B136] Oliveira A. C. Bertollo C. M. Rocha L. T. Nascimento E. B. Jr. Costa K. A. Coelho M. M. (2007). Antinociceptive and antiedematogenic activities of fenofibrate, an agonist of PPAR alpha, and pioglitazone, an agonist of PPAR gamma. Eur. J. Pharmacol. 561 (1-3), 194–201. 10.1016/j.ejphar.2006.12.026 17343847

[B137] Onuki M. Watanabe M. Ishihara N. Suzuki K. Takizawa K. Hirota M. (2019). A partial agonist for retinoid X receptor mitigates experimental colitis. Int. Immunol. 31 (4), 251–262. 10.1093/intimm/dxy089 30590577

[B138] Paterniti I. Impellizzeri D. Crupi R. Morabito R. Campolo M. Esposito E. (2013). Molecular evidence for the involvement of PPAR-delta and PPAR-gamma in anti-inflammatory and neuroprotective activities of palmitoylethanolamide after spinal cord trauma. J. Neuroinflamm. 10, 20. 10.1186/1742-2094-10-20 PMC3579707 23374874

[B139] Paukkeri E. L. Leppanen T. Sareila O. Vuolteenaho K. Kankaanranta H. Moilanen E. (2007). PPARalpha agonists inhibit nitric oxide production by enhancing iNOS degradation in LPS-treated macrophages. Br. J. Pharmacol. 152 (7), 1081–1091. 10.1038/sj.bjp.0707477 17891158 PMC2095111

[B140] Peng J. Zheng T. T. Li X. Liang Y. Wang L. J. Huang Y. C. (2019). Plant-Derived Alkaloids: The Promising Disease-Modifying Agents for Inflammatory Bowel Disease. Front. Pharmacol. 10, 351. 10.3389/fphar.2019.00351 31031622 PMC6473079

[B141] Peters J. M. Shah Y. M. Gonzalez F. J. (2012). The role of peroxisome proliferator-activated receptors in carcinogenesis and chemoprevention. Nat. Rev. Cancer 12 (3), 181–195. 10.1038/nrc3214 22318237 PMC3322353

[B142] Peters J. M. Gonzalez F. J. Muller R. (2015). Establishing the Role of PPARbeta/delta in Carcinogenesis. Trends Endocrinol. Metab. 26 (11), 595–607. 10.1016/j.tem.2015.09.004 26490384 PMC4631629

[B143] Peyrin-Biroulet L. Beisner J. Wang G. Nuding S. Oommen S. T. Kelly D. (2010). Peroxisome proliferator-activated receptor gamma activation is required for maintenance of innate antimicrobial immunity in the colon. Proc. Natl. Acad. Sci. U. S. A 107 (19), 8772–8777. 10.1073/pnas.0905745107 20421464 PMC2889363

[B144] Ponferrada A. Caso J. R. Alou L. Colon A. Sevillano D. Moro M. A. (2007). The role of PPARgamma on restoration of colonic homeostasis after experimental stress-induced inflammation and dysfunction. Gastroenterology 132 (5), 1791–1803. 10.1053/j.gastro.2007.02.032 17484875

[B145] Puleston J. Cooper M. Murch S. Bid K. Makh S. Ashwood P. (2005). A distinct subset of chemokines dominates the mucosal chemokine response in inflammatory bowel disease. Aliment Pharmacol. Ther. 21 (2), 109–120. 10.1111/j.1365-2036.2004.02262.x 15679760

[B146] Rahimian R. Zirak M. R. Keshavarz M. Fakhraei N. Mohammadi-Farani A. Hamdi H. (2016). Involvement of PPARgamma in the protective action of tropisetron in an experimental model of ulcerative colitis. Immunopharmacol. Immunotoxicol. 38 (6), 432–440. 10.1080/08923973.2016.1231202 27644482

[B147] Ramakers J. D. Verstege M. I. Thuijls G. Te Velde A. A. Mensink R. P. Plat J. (2007). The PPARgamma agonist rosiglitazone impairs colonic inflammation in mice with experimental colitis. J. Clin. Immunol. 27 (3), 275–283. 10.1007/s10875-007-9074-2 17510806 PMC1915631

[B148] Rao M. S. Reddy J. K. (2004). PPARalpha in the pathogenesis of fatty liver disease. Hepatology 40 (4), 783–786. 10.1002/hep.20453 15382158

[B149] Rezaie A. Kuenzig M. E. Benchimol E. I. Griffiths A. M. Otley A. R. Steinhart A. H. (2015). Budesonide for induction of remission in Crohn's disease. Cochrane Database Syst. Rev. 3 (6), CD000296. 10.1002/14651858.CD000296.pub4 PMC10613338 26039678

[B150] Riccardi L. Mazzon E. Bruscoli S. Esposito E. Crisafulli C. Di Paola R. (2009). Peroxisome proliferator-activated receptor-alpha modulates the anti-inflammatory effect of glucocorticoids in a model of inflammatory bowel disease in mice. Shock 31 (3), 308–316. 10.1097/SHK.0b013e31818339e7 18665053

[B151] Ricote M. Li A. C. Willson T. M. Kelly C. J. Glass C. K. (1998). The peroxisome proliferator-activated receptor-gamma is a negative regulator of macrophage activation. Nature 391 (6662), 79–82. 10.1038/34178 9422508

[B152] Riserus U. Sprecher D. Johnson T. Olson E. Hirschberg S. Liu A. (2008). Activation of peroxisome proliferator-activated receptor (PPAR)delta promotes reversal of multiple metabolic abnormalities, reduces oxidative stress, and increases fatty acid oxidation in moderately obese men. Diabetes 57 (2), 332–339. 10.2337/db07-1318 18024853

[B153] Rodriguez de Fonseca F. Navarro M. Gomez R. Escuredo L. Nava F. Fu J. (2001). An anorexic lipid mediator regulated by feeding. Nature 414 (6860), 209–212. 10.1038/35102582 11700558

[B154] Rousseaux C. Lefebvre B. Dubuquoy L. Lefebvre P. Romano O. Auwerx J. (2005). Intestinal antiinflammatory effect of 5-aminosalicylic acid is dependent on peroxisome proliferator-activated receptor-gamma. J. Exp. Med. 201 (8), 1205–1215. 10.1084/jem.20041948 15824083 PMC2213148

[B155] Saez E. Tontonoz P. Nelson M. C. Alvarez J. G. Ming U. T. Baird S. M. (1998). Activators of the nuclear receptor PPARgamma enhance colon polyp formation. Nat. Med. 4 (9), 1058–1061. 10.1038/2042 9734400

[B156] Sarraf P. Mueller E. Jones D. King F. J. DeAngelo D. J. Partridge J. B. (1998). Differentiation and reversal of malignant changes in colon cancer through PPARgamma. Nat. Med. 4 (9), 1046–1052. 10.1038/2030 9734398

[B157] Sartor R. B. (1997). Pathogenesis and immune mechanisms of chronic inflammatory bowel diseases. Am. J. Gastroenterol. 92 (12 Suppl), 5S–11S. 9395346

[B158] Savage S. R. McCollum G. W. Yang R. Penn J. S. (2015). RNA-seq identifies a role for the PPARbeta/delta inverse agonist GSK0660 in the regulation of TNFalpha-induced cytokine signaling in retinal endothelial cells. Mol. Vis. 21, 568–576. 26015769 PMC4443583

[B159] Scharl M. Huber N. Lang S. Furst A. Jehle E. Rogler G. (2015). Hallmarks of epithelial to mesenchymal transition are in Crohn's disease associated intestinal fibrosis. Clin. Transl. Med. 4, 1. 10.1186/s40169-015-0046-5 25852817 PMC4384762

[B160] Schoonjans K. Staels B. Auwerx J. (1996). The peroxisome proliferator activated receptors (PPARS) and their effects on lipid metabolism and adipocyte differentiation. Biochim. Biophys. Acta 1302 (2), 93–109. 10.1016/0005-2760(96)00066-5 8695669

[B161] Schreiber S. Heinig T. Thiele H. G. Raedler A. (1995). Immunoregulatory role of interleukin 10 in patients with inflammatory bowel disease. Gastroenterology 108 (5), 1434–1444. 10.1016/0016-5085(95)90692-4 7729636

[B162] Scirpo R. Fiorotto R. Villani A. Amenduni M. Spirli C. Strazzabosco M. (2015). Stimulation of nuclear receptor peroxisome proliferator-activated receptor-gamma limits NF-kappaB-dependent inflammation in mouse cystic fibrosis biliary epithelium. Hepatology 62 (5), 1551–1562. 10.1002/hep.28000 26199136 PMC4618241

[B163] Shanahan F. (2001). Probiotics in inflammatory bowel disease. Gut 48 (5), 609. 10.1136/gut.48.5.609 11302956 PMC1728287

[B164] Shen P. Zhang Z. He Y. Gu C. Zhu K. Li S. (2018). Magnolol treatment attenuates dextran sulphate sodium-induced murine experimental colitis by regulating inflammation and mucosal damage. Life Sci. 196, 69–76. 10.1016/j.lfs.2018.01.016 29355546

[B165] Shimizu M. Zhao Z. Ishimoto Y. Satsu H. (2009). Dietary taurine attenuates dextran sulfate sodium (DSS)-induced experimental colitis in mice. Adv. Exp. Med. Biol. 643, 265–271. 10.1007/978-0-387-75681-3_27 19239157

[B166] Solorzano C. Zhu C. Battista N. Astarita G. Lodola A. Rivara S. (2009). Selective N-acylethanolamine-hydrolyzing acid amidase inhibition reveals a key role for endogenous palmitoylethanolamide in inflammation. Proc. Natl. Acad. Sci. U. S. A 106 (49), 20966–20971. 10.1073/pnas.0907417106 19926854 PMC2791595

[B167] Speca S. Rousseaux C. Dubuquoy C. Rieder F. Vetuschi A. Sferra R. (2016). Novel PPARgamma Modulator GED-0507-34 Levo Ameliorates Inflammation-driven Intestinal Fibrosis. Inflammation Bowel Dis. 22 (2), 279–292. 10.1097/MIB.0000000000000618 PMC4718865 26535766

[B168] Storr M. A. Keenan C. M. Emmerdinger D. Zhang H. Yuce B. Sibaev A. (2008). Targeting endocannabinoid degradation protects against experimental colitis in mice: involvement of CB1 and CB2 receptors. J. Mol. Med. (Berl) 86 (8), 925–936. 10.1007/s00109-008-0359-6 18493729

[B169] Strober W. Fuss I. Mannon P. (2007). The fundamental basis of inflammatory bowel disease. J. Clin. Invest. 117 (3), 514–521. 10.1172/JCI30587 17332878 PMC1804356

[B170] Su C. G. Wen X. Bailey S. T. Jiang W. Rangwala S. M. Keilbaugh S. A. (1999). A novel therapy for colitis utilizing PPAR-gamma ligands to inhibit the epithelial inflammatory response. J. Clin. Invest. 104 (4), 383–389. 10.1172/JCI7145. 10449430 PMC408529

[B171] Suarez J. Romero-Zerbo Y. Marquez L. Rivera P. Iglesias M. Bermudez-Silva F. J. (2012). Ulcerative colitis impairs the acylethanolamide-based anti-inflammatory system reversal by 5-aminosalicylic acid and glucocorticoids. PloS One 7 (5), e37729. 10.1371/journal.pone.0037729 22662201 PMC3360619

[B172] Sugawara K. Olson T. S. Moskaluk C. A. Stevens B. K. Hoang S. Kozaiwa K. (2005). Linkage to peroxisome proliferator-activated receptor-gamma in SAMP1/YitFc mice and in human Crohn's disease. Gastroenterology 128 (2), 351–360. 10.1053/j.gastro.2004.11.001 15685547

[B173] Sun Y. Bennett A. (2007). Cannabinoids: a new group of agonists of PPARs. PPAR Res. 2007, 23513. 10.1155/2007/23513 18288264 PMC2220031

[B174] Sutherland T. E. Logan N. Ruckerl D. Humbles A. A. Allan S. M. Papayannopoulos V. (2014). Chitinase-like proteins promote IL-17-mediated neutrophilia in a tradeoff between nematode killing and host damage. Nat. Immunol. 15 (12), 1116–1125. 10.1038/ni.3023 25326751 PMC4338525

[B175] Takagi T. Naito Y. Tomatsuri N. Handa O. Ichikawa H. Yoshida N. (2002). Pioglitazone, a PPAR-gamma ligand, provides protection from dextran sulfate sodium-induced colitis in mice in association with inhibition of the NF-kappaB-cytokine cascade. Redox Rep. 7 (5), 283–289. 10.1179/135100002125000802 12688511

[B176] Takano H. Komuro I. (2009). Peroxisome proliferator-activated receptor gamma and cardiovascular diseases. Circ. J. 73 (2), 214–220. 10.1253/circj.cj-08-1071 19129679

[B177] Takeda T. Hosokawa M. Takeshita S. Irino M. Higuchi K. Matsushita T. (1981). A new murine model of accelerated senescence. Mech. Ageing Dev. 17 (2), 183–194. 10.1016/0047-6374(81)90084-1 7311623

[B178] Talley N. J. Abreu M. T. Achkar J. P. Bernstein C. N. Dubinsky M. C. Hanauer S. B. (2011). An evidence-based systematic review on medical therapies for inflammatory bowel disease. Am. J. Gastroenterol. 106 Suppl 1, S2–25; quiz S26. 10.1038/ajg.2011.58 21472012

[B179] Thomas A. W. Davies N. A. Moir H. Watkeys L. Ruffino J. S. Isa S. A. (2012). Exercise-associated generation of PPARgamma ligands activates PPARgamma signaling events and upregulates genes related to lipid metabolism. J. Appl. Physiol. (1985) 112 (5), 806–815. 10.1152/japplphysiol.00864.2011 22174394

[B180] Thomson A. B. Wright J. P. Vatn M. Bailey R. J. Rachmilewitz D. Adler M. (1995). Mesalazine (Mesasal/Claversal) 1.5 g b.d. vs. placebo in the maintenance of remission of patients with Crohn's disease. Aliment Pharmacol. Ther. 9 (6), 673–683. 10.1111/j.1365-2036.1995.tb00438.x 8824656

[B181] Tomas J. Mulet C. Saffarian A. Cavin J. B. Ducroc R. Regnault B. (2016). High-fat diet modifies the PPAR-gamma pathway leading to disruption of microbial and physiological ecosystem in murine small intestine. Proc. Natl. Acad. Sci. U. S. A 113 (40), E5934–E5943. 10.1073/pnas.1612559113 27638207 PMC5056107

[B182] Toyota Y. Nomura S. Makishima M. Hashimoto Y. Ishikawa M. (2017). Structure-activity relationships of rosiglitazone for peroxisome proliferator-activated receptor gamma transrepression. Bioorg. Med. Chem. Lett. 27 (12), 2776–2780. 10.1016/j.bmcl.2017.04.061 28465099

[B183] Travis S. P. Jewell D. P. (1994). Salicylates for ulcerative colitis–their mode of action. Pharmacol. Ther. 63 (2), 135–161. 10.1016/0163-7258(94)90042-6 7809176

[B184] Tsuboi K. Zhao L. Y. Okamoto Y. Araki N. Ueno M. Sakamoto H. (2007). Predominant expression of lysosomal N-acylethanolamine-hydrolyzing acid amidase in macrophages revealed by immunochemical studies. Biochim. Biophys. Acta 1771 (5), 623–632. 10.1016/j.bbalip.2007.03.005 17462942

[B185] Tsuboi K. Uyama T. Okamoto Y. Ueda N. (2018). Endocannabinoids and related N-acylethanolamines: biological activities and metabolism. Inflammation Regener. 38, 28. 10.1186/s41232-018-0086-5 PMC6166290 30288203

[B186] Vetter M. Neurath M. F. (2017). Emerging oral targeted therapies in inflammatory bowel diseases: opportunities and challenges. Therap. Adv. Gastroenterol. 10 (10), 773–790. 10.1177/1756283X17727388 PMC5638182 29051788

[B187] Vetuschi A. Pompili S. Gaudio E. Latella G. Sferra R. (2018). PPAR-gamma with its anti-inflammatory and anti-fibrotic action could be an effective therapeutic target in IBD. Eur. Rev. Med. Pharmacol. Sci. 22 (24), 8839–8848. 10.26355/eurrev_201812_16652 30575926

[B188] Viswakarma N. Jia Y. Bai L. Vluggens A. Borensztajn J. Xu J. (2010). Coactivators in PPAR-Regulated Gene Expression. PPAR Res. 2010, 250126. 10.1155/2010/250126 20814439 PMC2929611

[B189] Wada K. Nakajima A. Blumberg R. S. (2001). PPARgamma and inflammatory bowel disease: a new therapeutic target for ulcerative colitis and Crohn's disease. Trends Mol. Med. 7 (8), 329–331. 10.1016/S1471-4914(01)02076-7 11516972

[B190] Wang Y. X. Lee C. H. Tiep S. Yu R. T. Ham J. Kang H. (2003). Peroxisome-proliferator-activated receptor delta activates fat metabolism to prevent obesity. Cell 113 (2), 159–170. 10.1016/s0092-8674(03)00269-1 12705865

[B191] Wang Y. Parker C. E. Feagan B. G. MacDonald J. K. (2016a). Oral 5-aminosalicylic acid for maintenance of remission in ulcerative colitis. Cochrane Database Syst. Rev. 9 (5), CD000544. 10.1002/14651858.CD000544.pub4 27158764 PMC7045447

[B192] Wang Y. Parker C. E. Bhanji T. Feagan B. G. MacDonald J. K. (2016b). Oral 5-aminosalicylic acid for induction of remission in ulcerative colitis. Cochrane Database Syst. Rev. 4, CD000543. 10.1002/14651858.CD000543.pub4 27101467 PMC7045743

[B193] Wang L. Xie H. Xu L. Liao Q. Wan S. Yu Z. (2017). rSj16 Protects against DSS-Induced Colitis by Inhibiting the PPAR-alpha Signaling Pathway. Theranostics 7 (14), 3446–3460. 10.7150/thno.20359 28912887 PMC5596435

[B194] Wang Z. Koonen D. Hofker M. Bao Z. (2018). 5-aminosalicylic acid improves lipid profile in mice fed a high-fat cholesterol diet through its dual effects on intestinal PPARgamma and PPARalpha. PloS One 13 (1), e0191485. 10.1371/journal.pone.0191485 29352300 PMC5774772

[B195] Williams C. Panaccione R. Ghosh S. Rioux K. (2011). Optimizing clinical use of mesalazine (5-aminosalicylic acid) in inflammatory bowel disease. Therap. Adv. Gastroenterol. 4 (4), 237–248. 10.1177/1756283X11405250 PMC3131170 21765868

[B196] Willson T. M. Lambert M. H. Kliewer S. A. (2001). Peroxisome proliferator-activated receptor gamma and metabolic disease. Annu. Rev. Biochem. 70, 341–367. 10.1146/annurev.biochem.70.1.341 11395411

[B197] Wynn T. A. (2008). Cellular and molecular mechanisms of fibrosis. J. Pathol. 214 (2), 199–210. 10.1002/path.2277 18161745 PMC2693329

[B198] Xu H. E. Lambert M. H. Montana V. G. Parks D. J. Blanchard S. G. Brown P. J. (1999). Molecular recognition of fatty acids by peroxisome proliferator-activated receptors. Mol. Cell 3 (3), 397–403. 10.1016/s1097-2765(00)80467-0 10198642

[B199] Yamamoto-Furusho J. K. Penaloza-Coronel A. Sanchez-Munoz F. Barreto-Zuniga R. Dominguez-Lopez A. (2011). Peroxisome proliferator-activated receptor-gamma (PPAR-gamma) expression is downregulated in patients with active ulcerative colitis. Inflammation Bowel Dis. 17 (2), 680–681. 10.1002/ibd.21322 20848495

[B200] Yamamoto-Furusho J. K. Jacintez-Cazares M. Furuzawa-Carballeda J. Fonseca-Camarillo G. (2014). Peroxisome proliferator-activated receptors family is involved in the response to treatment and mild clinical course in patients with ulcerative colitis. Dis. Markers 2014, 932530. 10.1155/2014/932530 25548431 PMC4274912

[B201] Yamazaki K. Takazoe M. Tanaka T. Kazumori T. Nakamura Y. (2002). Absence of mutation in the NOD2/CARD15 gene among 483 Japanese patients with Crohn's disease. J. Hum. Genet. 47 (9), 469–472. 10.1007/s100380200067 12202985

[B202] Yamazaki K. Onouchi Y. Takazoe M. Kubo M. Nakamura Y. Hata A. (2007). Association analysis of genetic variants in IL23R, ATG16L1 and 5p13.1 loci with Crohn's disease in Japanese patients. J. Hum. Genet. 52 (7), 575–583. 10.1007/s10038-007-0156-z 17534574

[B203] Yao J. Lu Y. Zhi M. Hu P. Wu W. Gao X. (2017). Dietary n3 polyunsaturated fatty acids ameliorate Crohn's disease in rats by modulating the expression of PPARgamma/NFAT. Mol. Med. Rep. 16 (6), 8315–8322. 10.3892/mmr.2017.7673 28990050

[B204] Yu Y. Zhu W. Liang Q. Liu J. Yang X. Sun G. (2018). Tropisetron attenuates lipopolysaccharide induced neuroinflammation by inhibiting NF-kappaB and SP/NK1R signaling pathway. J. Neuroimmunol. 320, 80–86. 10.1016/j.jneuroim.2018.05.001 29759144

[B205] Zhang M. Sun K. Wu Y. Yang Y. Tso P. Wu Z. (2017). Interactions between Intestinal Microbiota and Host Immune Response in Inflammatory Bowel Disease. Front. Immunol. 8, 942. 10.3389/fimmu.2017.00942 28855901 PMC5558048

[B206] Zhang W. Cheng C. Han Q. Chen Y. Guo J. Wu Q. (2019). Flos Abelmoschus manihot extract attenuates DSS-induced colitis by regulating gut microbiota and Th17/Treg balance. Biomed. Pharmacother. 117, 109162. 10.1016/j.biopha.2019.109162 31254739

[B207] Zhao L. Zhang S. He P. (2017). Mechanistic Understanding of Herbal Therapy in Inflammatory Bowel Disease. Curr. Pharm. Des. 23 (34), 5173–5179. 10.2174/1381612823666171010124414 29032748

[B208] Zheng J. Corzo C. Chang M. R. Shang J. Lam V. Q. Brust R. (2018). Chemical Crosslinking Mass Spectrometry Reveals the Conformational Landscape of the Activation Helix of PPARgamma; a Model for Ligand-Dependent Antagonism. Structure 26 1431-1439 (11), e1436. 10.1016/j.str.2018.07.007 PMC6221991 30146169

[B209] Zhou M. He J. Shen Y. Zhang C. Wang J. Chen Y. (2017). New Frontiers in Genetics, Gut Microbiota, and Immunity: A Rosetta Stone for the Pathogenesis of Inflammatory Bowel Disease. BioMed. Res. Int. 2017, 8201672. 10.1155/2017/8201672 28831399 PMC5558637

